# Peripheral serum metabolomic profiles inform central cognitive impairment

**DOI:** 10.1038/s41598-020-70703-w

**Published:** 2020-08-20

**Authors:** Jingye Wang, Runmin Wei, Guoxiang Xie, Matthias Arnold, Alexandra Kueider-Paisley, Gregory Louie, Siamak Mahmoudian Dehkordi, Colette Blach, Rebecca Baillie, Xianlin Han, Philip L. De Jager, David A. Bennett, Rima Kaddurah-Daouk, Wei Jia

**Affiliations:** 1 University of Hawaii Cancer Center, 701 Ilalo Street, Honolulu, HI 96813 USA; 2 Department of Molecular Biosciences and Bioengineering, University of Hawaii at Manoa, Honolulu, HI USA; 3 Department of Psychiatry and Behavioral Sciences, Duke University, Durham, NC USA; 4 Institute of Computational Biology, Helmholtz Zentrum München, German Research Center for Environmental Health, Neuherberg, Germany; 5 Duke Molecular Physiology Institute, Duke University, Durham, NC USA; 6 Rosa & Co LLC, San Carlos, CA USA; 7 University of Texas Health Science Center at San Antonio, San Antonio, TX USA; 8 Center for Translational & Computational Neuroimmunology, Columbia University College of Physicians and Surgeons Department of Neurology, New York, NY USA; 9 Rush Alzheimer’s Disease Center, Rush University Medical Center, Chicago, IL USA; 10 Institute of Brain Sciences, Duke University, Durham, NC USA; 11 Department of Medicine, Duke University, Durham, NC USA

**Keywords:** Predictive markers, Alzheimer's disease

## Abstract

The incidence of Alzheimer's disease (AD) increases with age and is becoming a significant cause of worldwide morbidity and mortality. However, the metabolic perturbation behind the onset of AD remains unclear. In this study, we performed metabolite profiling in both brain (n = 109) and matching serum samples (n = 566) to identify differentially expressed metabolites and metabolic pathways associated with neuropathology and cognitive performance and to identify individuals at high risk of developing cognitive impairment. The abundances of 6 metabolites, glycolithocholate (GLCA), petroselinic acid, linoleic acid, myristic acid, palmitic acid, palmitoleic acid and the deoxycholate/cholate (DCA/CA) ratio, along with the dysregulation scores of 3 metabolic pathways, primary bile acid biosynthesis, fatty acid biosynthesis, and biosynthesis of unsaturated fatty acids showed significant differences across both brain and serum diagnostic groups (*P*-value < 0.05). Significant associations were observed between the levels of differential metabolites/pathways and cognitive performance, neurofibrillary tangles, and neuritic plaque burden. Metabolites abundances and personalized metabolic pathways scores were used to derive machine learning models, respectively, that could be used to differentiate cognitively impaired persons from those without cognitive impairment (median area under the receiver operating characteristic curve (AUC) = 0.772 for the metabolite level model; median AUC = 0.731 for the pathway level model). Utilizing these two models on the entire baseline control group, we identified those who experienced cognitive decline in the later years (AUC = 0.804, sensitivity = 0.722, specificity = 0.749 for the metabolite level model; AUC = 0.778, sensitivity = 0.633, specificity = 0.825 for the pathway level model) and demonstrated their pre-AD onset prediction potentials. Our study provides a proof-of-concept that it is possible to discriminate antecedent cognitive impairment in older adults before the onset of overt clinical symptoms using metabolomics. Our findings, if validated in future studies, could enable the earlier detection and intervention of cognitive impairment that may halt its progression.

## Introduction

Alzheimer's disease (AD), one of the top 10 leading causes of death in the United States, is an increasing challenge for health care systems and will result in increased economic burden as increasing numbers of new cases are diagnosed annually^1,2^. Currently, there is no therapy to prevent or slow AD progression, which may be due to the inability to detect AD before its progression into evident cognitive decline. Identification of early biomarkers associated with preclinical symptoms would allow early intervention or preventive strategies to be developed^3^. Research has identified multiple neurochemical perturbations in AD, including amyloid precursor protein metabolism, phosphorylation of tau protein, and a wide range of metabolic perturbations^4^. Unfortunately, current biomarkers for early disease, including cerebrospinal fluid (CSF) beta-amyloid and tau levels^5^, structural and functional magnetic resonance imaging^6^, the recent use of brain amyloid imaging^7^ or inflammaging^8^, and CSF markers to track brain atrophy and deposition of cortical beta-amyloid and neurofibrillary tangles, are limited because they are either invasive, time-consuming or expensive.

Recent studies have focused on obtaining biomarkers to identify features that differentiate persons with cognitive impairment from persons without cognitive impairment. Molecular markers sensitive to the underlying pathogenic factors would be highly relevant to early disease detection and facilitation of disease monitoring and treatment responses. Metabolomics is an unbiased approach to study small-molecule metabolites that offers hope for the discovery of more biomarkers for AD. This profiling technology has already been used to identify differential metabolites that can distinguish mild cognitive impairment (MCI) subjects who will develop AD from stable MCI^9^. Mounting evidence suggests that AD is closely accompanied with the abnormal bile acid (BA) metabolism^10–13^, free fatty acid (FFA) metabolism^14–16^, lipid metabolism^17,18^, and neurotransmitter metabolism^19^. BAs have become increasingly recognized as important metabolic signaling molecules that modulate lipid, glucose, and energy metabolism^20^. More importantly, BAs in brain act as neuroactive steroids^21^. Different classes of BAs can either inhibit or potentiate GABA_α_, and inhibit NMDA receptors while also exerting neuroprotective effects ^22,23^. Recent cross-sectional studies have shown differences in blood BAs in AD compared with non-cognitively impaired individuals^24,25^. Additionally, researchers found an accumulation of FFAs in the hippocampus and cortex of AD mice compared to control mice^26,27^. Another animal study examined the role of elevated FFA in the pathogenesis of AD and established a potential mechanism of FFA causing hyperphosphorylation of tau through astroglia-mediated oxidative stress^28^. Alterations of FFAs have also been detected in postmortem AD brains tissues^14^ and serum samples^16^, which may indicate an alternative fuel source before the onset of clinical symptoms^29^. These observations have given rise to the possibility that metabolic perturbations could presage the onset of cognitive impairment and therefore aid in the identification of individuals with higher risks by providing additional information to use with standard clinical markers.

In this study, we performed metabolomic profiling in participants from a large, longitudinal cohort, with the goal of identifying metabolic changes as well as key metabolic pathways that might serve as new predictors of future cognitive impairment in older adults.

## Materials and methods

### Participants

The Religious Orders Study (ROS), which began in 1994, is a longitudinal clinical-pathologic cohort study of risk factors of cognitive decline and incident dementia run from the Rush Alzheimer’s Disease Center that is comprised of individuals from religious communities (e.g., Catholic brothers, nuns, and priests) across the USA^30,31^. The Rush Memory and Aging Project (MAP), which began in 1997 includes participants from northeastern Illinois, USA with a broader range of socioeconomic status and life experiences^31^. Participants in both studies enroll without known dementia, agree to annual clinical evaluation, and organ donation. Both studies were approved by an Intuitional Review Board of Rush University Medical Center. All subjects signed an informed consent, an Anatomic Gift Act, and a repository consent to allow their biospecimens and data to be used for ancillary studies. All research was performed in accordance with relevant guidelines/regulations set forth by the Rush University Medical Center. Both studies are conducted by the same team of examiners and share a large common core of data collection at the item level to allow for efficient merging of data.

### Cognitive performance tests

Cognitive performance was measured using a battery of 19 cognitive performance tests, 17 of which could be summarized in five cognitive domains (i.e., episodic memory, working memory, semantic memory, perceptual orientation/visuospatial ability, and perceptual speed) (Table S1). Domains are created by averaging the z-scores, based on mean and standard deviation from all baseline data, for tests in each domain. The global cognitive function score is calculated by averaging z-scores for all 17 tests to yield a global measure of cognitive function. Additionally, the Mini-Mental State Examination was also administered to characterize the cohort.

### Clinical diagnoses

Medical conditions were documented via self-report and clinical evaluation. Clinical diagnoses each year were determined blinded to previously collected data. A three-step process starts with an actuarial decision tree based on the history of cognitive decline, and impairment ratings in five cognitive domains based on cutoffs for 11 cognitive tests^32^ followed by clinical judgment by a neuropsychologist for cognitive impairment and determination of dementia and its causes by a clinician (i.e., neurologist, geriatrician, second neuropsychologist, geriatric nurse practitioner)^33^. The diagnosis of AD follows the criteria of the National Institute of Neurological and Communicative Disorders and Stroke and the Alzheimer’s Disease and Related Disorders Association (NINCDS/ADRDA)^34^. Participants were categorized as (a) AD, (b) MCI if diagnosed cognitive impairment by the neuropsychologist but not diagnosed dementia by the clinician^32^, and (c) no cognitive impairment (NCI) if diagnosed without AD or MCI^35^. At the time of death, brain autopsies and histopathological exams were performed by clinicians to confirm the diagnosis. After an autopsy was completed, a spectrum of neuropathologic diagnoses was obtained, such as a pathologic diagnosis of AD as defined using the modified NIA Reagan criteria. However, many other pathologies were present in the brains of older individuals (the mean age of death is 88.8 years old in ROSMAP), and they were catalogued for each participant.

NCI converters were those participants who were cognitively normal at the time of blood draw and then experienced the cognitive decline (MCI or AD) at the time of death, while NCI non-converters were participants who remained cognitively normal during follow-up.

### Neuropathology

Upon death, a postmortem neuropathological evaluation was implemented, and the procedures follow those outlined by the pathologic dataset recommended by the National Alzheimer’s Disease Coordinating Center. Brains of deceased subjects were removed, weighed, cut into one cm-thick coronal slabs and stored. Each brain was examined for the neuropathological indices of common pathologies that contribute to cognitive impairment. The location, age, and volume of all macroscopic infarcts were recorded, and tissue was obtained for histological confirmation, in addition to the identification of microscopic infarctions, as previously described^36,37^. AD pathology was identified using the modified Bielschowsky silver stain technique and by use of the Consortium to Establish a Registry for Alzheimer’s Disease (CERAD) criteria^38^ and NIA-Reagan criteria^39^, while the assessment of neurofibrillary tangles was based on Braak criteria^40^ as described previously^41^. The CERAD score, a semi-quantitative measure of neuritic plaque burden, is made of 4 levels: 4 = no AD, 3 = possible AD, 2 = probable AD and 1 = definite AD. As recommended, CERAD scores were reclassified to a binary level: score 1 ~ 2, score 3 ~ 4. Seven categories of Braak stages were based on the region and severity of neurofibrillary tangles pathology.

### Metabolites quantification

Using targeted metabolomics protocols^42^ and profiling protocols^43^ established in previous studies, BAs were quantified by ultra-performance liquid chromatography triple quadrupole mass spectrometry (UPLC-TQMS) (Waters XEVO TQ-S, Milford, USA) and other metabolites were quantified by gas chromatography time-of-flight mass spectrometry (GC-TOFMS) (Leco Corporation, St Joseph, USA). Details are described in the Supporting Information.

### Statistical analysis

Stratifying by clinical diagnosis, continuous demographic variables were expressed as mean [standard deviation (SD)] and tested by Wilcoxon rank-sum test, while categorical demographic variables were expressed as n (percentage) and tested by Chi-square test. Missing values in quantitative metabolites due to limits of quantification were regarded as left-censored missing and imputed by GSimp^44,45^. Individual BA concentrations were normalized to the total BAs concentration (i.e., the proportion of total BAs). Metabolites were reported as median (25% quantile, 75% quantile) and tested by univariate analysis (Wilcoxon rank-sum test). Due to the limited sample size of the AD group (11 participants) in serum samples, we combined MCI and AD participants into an aggregate group (MCI/AD) for the following data analysis. Log-transformed abundances were used in the following data analysis. We additionally generated 12 BA ratios based on the BA metabolic pathway topology.

To identify metabolites differentially expressed in participants with cognitive decline, we used ordinal logistic regression to compare metabolites across three groups (NCI, MCI, AD) for brain samples and logistic regression across two groups (NCI, MCI/AD) for serum samples. To control the positive false discovery rate, *Q*-values were calculated based on *P*-values. Additionally, for serum samples, we adjusted for potential confounders, (e.g., fasting status, supplements, diabetic and lipid lowering medications) using logistic regressions. The relationships between log-transformed brain metabolites levels with neurofibrillary tangle burden and neuritic plaque burden were expressed as boxplots across Braak scores (Kruskal–Wallis test) and CERAD scores (Wilcoxon rank-sum test), respectively. Using Spearman’s rank correlation test, we further evaluated the associations between the abundances of each identified metabolite and the global cognitive function score in both brain and serum samples. Linear regression models with each individual metabolite used as the predictor and each cognitive test as the response variable (adjusted for age, gender, years of education, and presence of APOE ε4) were used to test the associations between metabolite and cognitive function. Similar analyses with an additional adjustment of BMI were conducted for serum samples. The Wilcoxon rank-sum test was carried out to explore whether identified variables were differentially expressed between NCI (converters) vs. NCI (non-converters), and between NCI (converters) vs. MCI/AD in sera. Then, we built a random forest (RF) predictive model to differentiate NCI (non-converters) vs. MCI/AD using glycolithocholate (GLCA), deoxycholate/cholate (DCA/CA) ratio, petroselinic acid, linoleic acid, myristic acid, palmitic acid, palmitoleic acid, and age as the predictors.

To differentiate MCI/AD vs. NCI (non-converters), we randomly split the data into 70% (training set) and 30% (testing set) 100 times. Each time, we trained an RF model on the training set to differentiate the MCI/AD from NCI (non-converters) and evaluated it on the testing set using the area under the receiver operating characteristic curve (AUROC), sensitivity (SE) and specificity (SP). A final model was built on the whole NCI (non-converters) and MCI/AD data.

To investigate the pre-clinical predictive potentials as well as to validate the classification performance of our model, we utilized this model on the entire baseline NCI group to identify those NCI (converters) from NCI (non-converters). The differences of RF scores between NCI (non-converters) vs. NCI (converters), and NCI (non-converters) vs. MCI/AD groups were tested by the Wilcoxon rank-sum test. To determine whether RF scores could independently differentiate NCI (converters) from NCI (non-converters) in the presence of potential confounders, we used the logistic regression method with RF scores as the predictor adjusting for gender, years of education, APOE ε4, and BMI. Additionally, we fit linear mixed effects models to evaluate correlations between RF scores with global cognitive function and each of the five cognitive domains separately with a random effects term for education and BMI and fixed effects terms for RF score, gender, and APOE ε4.

For the personalized pathway level analyses, we extracted metabolite information from the Human Metabolome Database (HMDB)^46^ and metabolic pathway information from the Kyoto Encyclopedia of Genes and Genomes (KEGG) database^47^ to map affiliated metabolites to metabolic pathways. We used the *pathifier* algorithm^48^ to transfer metabolic level information of each sample to pathway level by generating a pathway dysregulation score (PDS). For each pathway, each sample was projected onto a directed principal curve^49^, which was yielded depending on leading principal components of the pathway, to optimally pass through the cloud of samples. PDS was the distance along the curve between the projection of each sample and that of NCI. Thus, PDS could capture the pathway-level extent of abnormality (increments or decrements) for each participant relative to those with NCI. We performed similar data analysis on pathway level data to what we did on metabolomics level data. We tried to identify differential pathways using ordinal logistic regression across NCI, MCI, and AD groups in brain samples and logistic regression for NCI and MCI/AD in serum samples. Next, we explored the associations between identified pathways with neuropathology (Kruskal–Wallis test for Braak scores, Wilcoxon rank-sum test for CERAD scores) and cognitive performance (Spearman’s rank correlation test for the global cognitive function, linear regression with adjustments for each cognitive test). Then, we examined the predictive potential of identified pathways in serum samples using univariate analysis (Wilcoxon rank-sum test for NCI (converters) vs. NCI (non-converters), NCI (converters) vs. MCI/AD). Finally, we built RF models on 70% training sets and tested them on 30% testing sets according to 100 times random splitting on the model construction data, and applied the final model on the validation data using ROC, SE, SP as evaluation methods. The overall workflow chart of the data and the analysis are shown in Fig. S9.

Data were analyzed using R version 3.5.1 with packages including *pROC*, *pathifier*, *randomForest*, *ggplot2*, *ggsignif*, and *MASS*. The statistically significance was determined by a threshold of unadjusted *P*-values < 0.05 and *Q*-values < 0.2^50^.

## Results

### Participants and characteristics

For the joint analyses of the ROS/MAP study, we measured metabolomics of 566 serum samples (446 NCI, 109 MCI, and 11 AD at the blood draw) and 109 postmortem brain tissues from dorsolateral prefrontal cortex (51 NCI, 31 MCI, and 27 AD at the time of death). Among 109 brain samples and 566 serum samples, a total of 92 participants had both brain and blood metabolomics data. NCI participants (n = 466) were further categorized into 90 “NCI (converters)” and 356 “NCI (non-converters)”. NCI (converters) were those participants who were cognitively normal (NCI) at the time of blood draw and then experienced the cognitive decline (MCI or AD) at the time of death, while NCI (non-converters) were participants who remained cognitively normal during follow-up. Among 446 NCI participants, the time between the blood draw and conversion ranged from 0 to 19 years with a median of 3 years (Fig. S10). Detailed demographic characteristics of the serum samples and postmortem brain samples are included in Table 1. Among participants with postmortem brain samples, AD patients tended to have at least one APOE ε4 allele compared to the NCI group as expected. The mean age of NCI and MCI/AD group at the time of blood draw among serum samples was 80.77 years (SD: 7.37) and 86.30 (SD: 6.26), respectively. Similarly, the age and the percentage of APOE ε4 carriers were higher in the NCI (converters) group than the NCI (non-converters) group. We did not observe other significant demographic characteristics differences across clinical groups (Table 1).

**Table 1:** Detailed demographic characteristics of study samples.

	Overall	NCI	MCI	AD
**Brain samples**				
*N*	109	51	31	27
Age, mean (SD)	90.40 (5.86)	90.06 (5.56)	90.72 (6.14)	90.68 (6.28)
Male, *n* (%)	31 (28.4)	18 (35.3)	9 (29.0)	4 (14.8)
Education, mean (SD)	15.08 (3.17)	15.25 (3.60)	14.65 (2.27)	15.26 (3.23)
APOE ε4-carrier, *n* (%)	22 (21.2)	6 (12.2)	7 (24.1)	9 (34.6)*

	Overall	NCI	MCI/AD	
**Serum samples**				
*N*	566	446	120	
Age (years), mean (SD)	81.94 (7.49)	80.77 (7.37)	86.30 (6.26)**	
Male, *n* (%)	121 (21.4)	93 (20.9)	28 (23.3)	
Education (years), mean (SD)	15.71 (3.11)	15.79 (3.12)	15.42 (3.09)	
APOE ε4-carrier, *n* (%)	95 (19.9)	68 (18.1)	27 (26.5)	

	Overall	NCI (non-converters)	NCI (converters)	
**Serum NCI samples**				
*N*	446	356	90	
Age (years), mean (SD)	80.77 (7.37)	79.73 (7.42)	84.87 (5.54)***	
Male, *n* (%)	93 (20.9)	74 (20.8)	19 (21.1)	
Education (years), mean (SD)	15.79 (3.12)	15.87 (3.16)	15.48 (2.94)	
APOE ε4-carrier, *n* (%)	68 (18.1)	43 (14.8)	25 (29.4)****	

*Chi-square test, *P*-value < 0.05 comparing AD vs. NCI.

**Wilcoxon rank sum test, *P*-value < 0.05 comparing MCI/AD vs. NCI.

***Wilcoxon rank sum test, *P*-value < 0.05 comparing NCI (converters) vs. NCI (non-converters).

****Chi-square test, *P*-value < 0.05 comparing NCI (converter) vs. NCI (non-converter.

*NCI* cognitively normal, *MCI* mild cognitive impairment, *AD* Alzheimer’s disease, *APOE ε4* apolipoprotein E epsilon 4 allele, *SD* standard deviation.

### Identifying metabolites differentially expressed in participants with cognitive impairment

**Figure 1:**
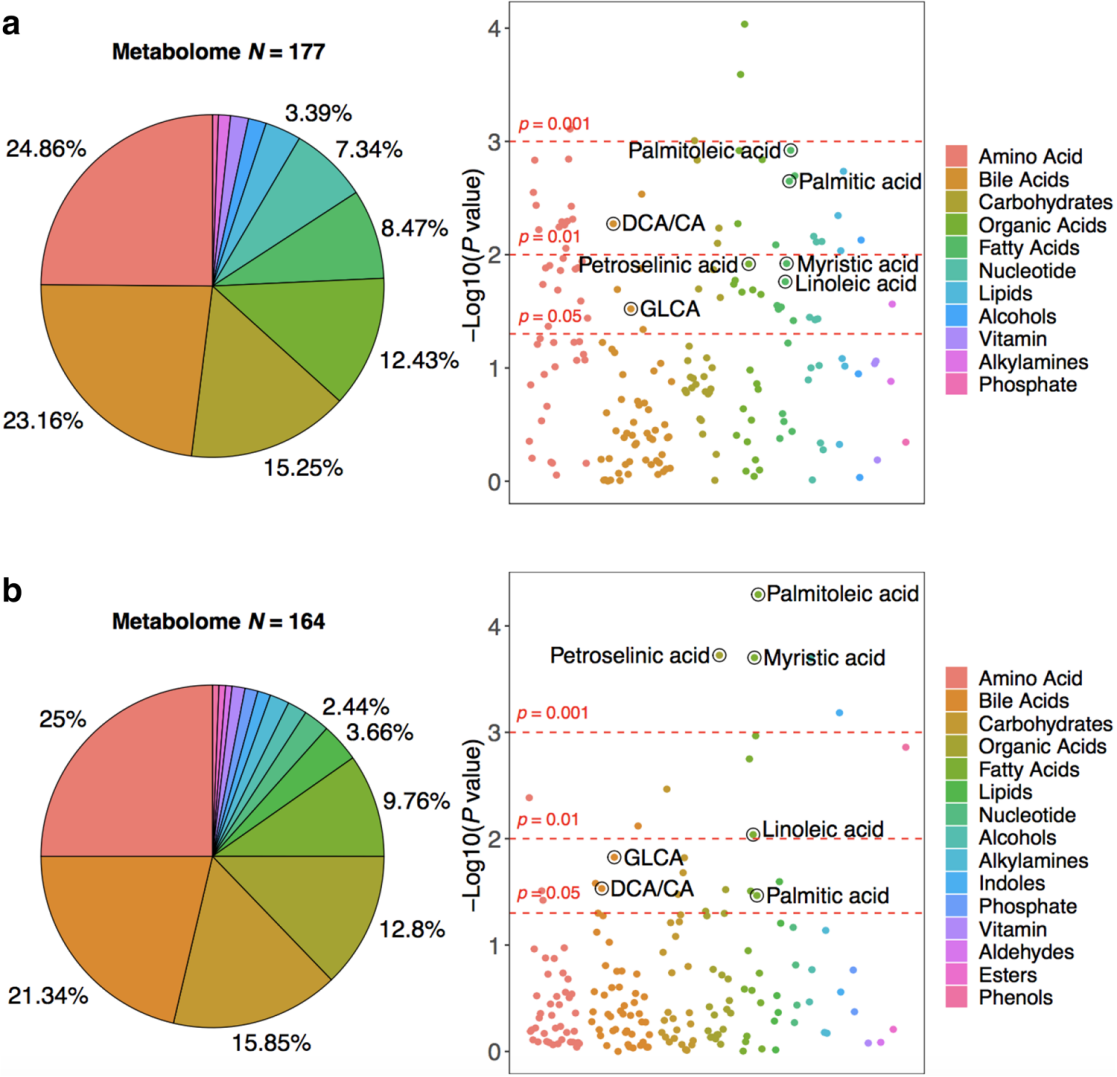
Brain metabolome and serum metabolome composition and alterations. (**a**) Left panel: the brain metabolome composition. Right panel: – log10 (*P*-value) across clinical groups of brain tissues (NCI, MCI, AD). (**b**) Left panel: the serum metabolome composition. Right panel: – log10 (*P*-value) across clinical groups of serum tissues (NCI, MCI/AD).

In this study, 177 metabolites and 164 metabolites (129 overlapping metabolites) were detected in brain tissues and serum samples, respectively (Tables S7, S8). Amino acids, BAs, carbohydrates, organic acids, and fatty acids were the predominant types of annotated metabolites (accounting for 84.17% of all the metabolites in brain tissues, and 84.75% in serum samples) (Fig. 1a,b, left panel). A total of seven metabolites (1 BA, 1 BA ratio, 1 organic acid known as a long-chain fatty acid, 4 fatty acids) showed significant differences across clinical groups in both brain and serum samples (*P*-value < 0.05 and *Q*-value < 0.2, ordinal logistic regression for brain samples, logistic regression for serum samples) (Fig. 1a,b, right panel, Tables S14, S15). After adjusting for confounders (i.e. fasting status, supplement use, diabetic and lipid lowering medications), most of serum metabolites remained statistically significant (Table S18). In brain tissues, increments of the levels of GLCA, DCA/CA ratio, petroselinic acid, linoleic acid, myristic acid, palmitic acid, and palmitoleic acid followed the pattern NCI < MCI < AD. We observed increments of GLCA, DCA/CA ratio and decrements of petroselinic acid, linoleic acid, myristic acid, palmitic acid, palmitoleic acid in sera of MCI/AD compared to controls (Table 2).

**Table 2:** Levels of metabolites differentially expressed in participants by diagnostic group.

	NCI	MCI	AD
**Brain samples**			
GLCA %, median [IQR]	0.44 [0.24, 0.84]	0.59 [0.25, 1.22]	0.86 [0.42, 1.24]*
DCA/CA, median [IQR]	6.06 [2.57, 11.79]	9.14 [6.00, 15.75]	8.60 [4.26, 31.96]*
Petroselinic acid, median [IQR]	1696.67 [1,323.38, 2,166.37]	1856.55 [1,529.43, 2,374.30]	2,181.55 [1,761.35, 2,617.07]**
Linoleic acid, median [IQR]	13.86 [10.64, 18.15]	14.37 [10.40, 21.62]	19.93 [15.08, 29.43]***
Myristic acid, median [IQR]	164.14 [130.79, 205.57]	177.10 [136.34, 205.38]	206.86 [177.15, 246.65]**
Palmitic acid, median [IQR]	8,504.53 [6,898.86, 10,496.19]	9,025.76 [7,811.72, 10,964.45]	10,323.59 [9,204.13, 12,785.95]***
Palmitoleic acid, median [IQR]	64.96 [48.13, 79.72]	75.39 [61.45, 100.90]	82.06 [64.21, 127.76]**

	NCI	MCI/AD	
**Serum samples**			
GLCA %, median [IQR]	0.96 [0.49, 1.60]	1.10 [0.54, 1.94]	
DCA/CA, median [IQR]	10.30 [3.11, 28.10]	14.78 [3.69, 44.75]^#^	
Petroselinic acid, median [IQR]	0.84 [0.45, 1.41]	0.49 [0.33, 1.02]^###^	
Linoleic acid, median [IQR]	0.90 [0.64, 1.24]	0.73 [0.58, 0.99]^##^	
Myristic acid, median [IQR]	0.84 [0.59, 1.22]	0.63 [0.52, 0.97]^###^	
Palmitic acid, median [IQR]	0.89 [0.68, 1.18]	0.78 [0.62, 1.00]^##^	
Palmitoleic acid, median [IQR]	0.75 [0.35, 1.35]	0.43 [0.23, 0.87]^###^	

*P-value < 0.05, **P-value < 0.01, ***P-value < 0.001, by Wilcoxon rank sum test comparing AD vs. NCI.

^#^*P*-value < 0.05, ^##^*P*-value < 0.01, ^###^*P*-value < 0.001, by Wilcoxon rank sum test comparing MCI/AD vs. NCI.

*NCI* cognitively normal, *MCI* mild cognitive impairment, *AD* Alzheimer’s disease, *IQR* interquartile range.

The trend of increments of identified metabolites in brain samples, increments of BAs and decrements of FFAs in serum samples were further validated within 92 individuals with both brain and serum samples. From NCI to MCI and AD groups, increments of identified metabolites were observed in brain samples (Table S11). The increasing trend of GLCA, DCA/CA ratio and decreasing trend of FFAs among MCI/AD group relative to NCI group were detected in sera (Table S11).

**Figure 2:**
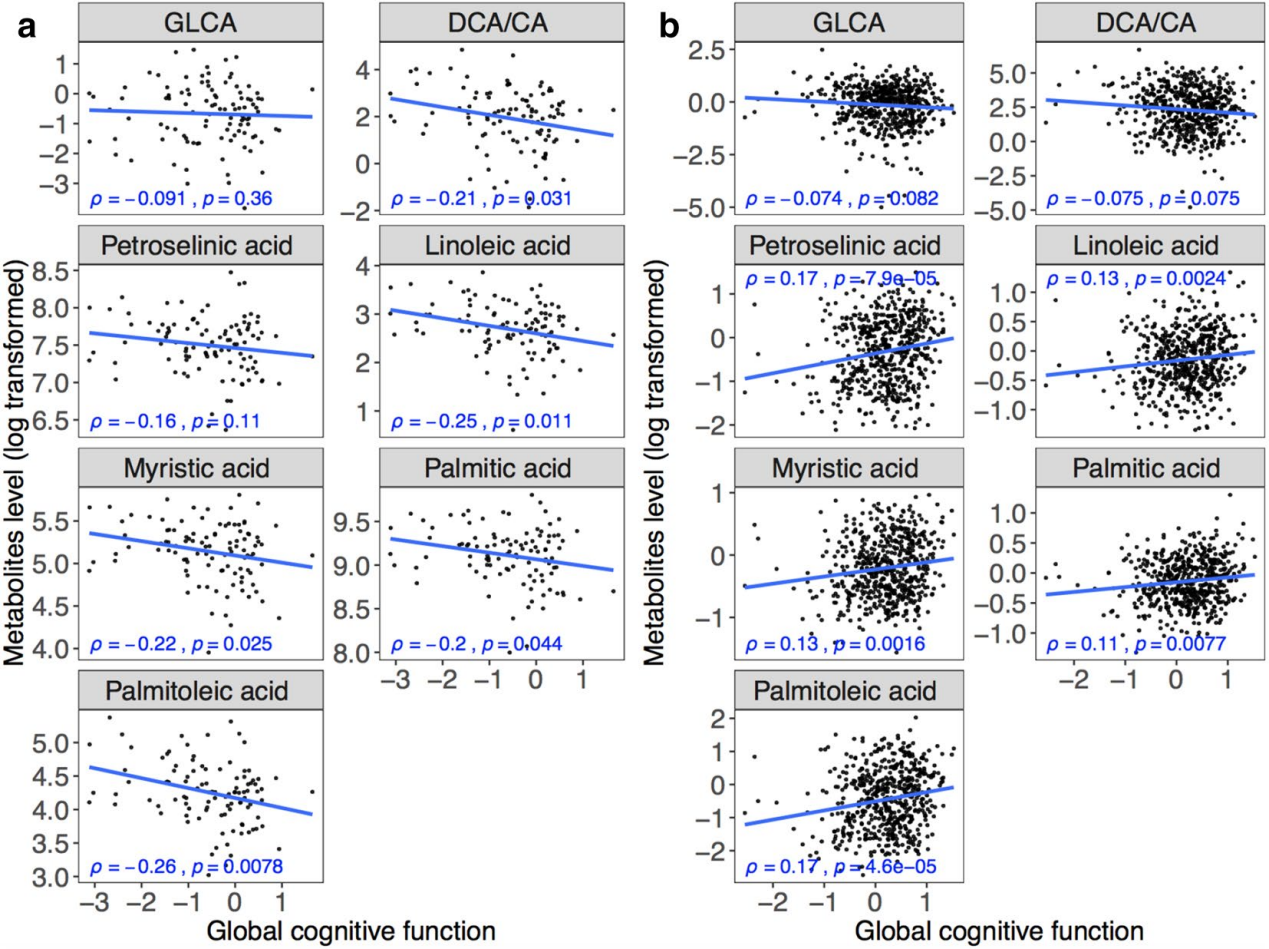
Associations between metabolites level and global cognitive function. (**a**) Boxplots showing group differences and *P* values for identified metabolites across Braak groups for brain tissue abundances. (**b**) Boxplots showing group differences and significances for identified metabolites across CERAD groups for brain tissue abundances. ρ, correlation coefficient of Spearman’s rank correlation test.

The seven brain metabolites were all negatively correlated with global cognitive function where higher scores indicate better cognitive performance (ρ = − 0.091 for GLCA; ρ = − 0.21 for DCA/CA ratio, ρ = − 0.16 for petroselinic acid, ρ = − 0.25 for linoleic acid, ρ = − 0.22 for myristic acid, ρ = − 0.2 for palmitic acid, and ρ = − 0.26 for palmitoleic acid) using Spearman’s rank correlation analysis (Fig. 2a). Similarly, after adjusting for age, gender, years of education, and APOE ε4, all identified metabolites remained negatively correlated with tests in five cognitive domains and the Mini-Mental State Exam (see Table S2 for significant correlation pairs). The serum concentration of two BAs showed negative correlations with global cognitive function (ρ = − 0.074 for GLCA; ρ = − 0.075 for DCA/CA ratio), conversely fatty acids demonstrated positive correlations (ρ = 0.17 for petroselinic acid, ρ = 0.13 for linoleic acid, ρ = 0.13 for myristic acid, ρ = 0.11 for palmitic acid, and ρ = 0.17 for palmitoleic acid) (Fig. 2b). Linear regression revealed similar, consistent results in serum samples with adjustment for age, gender, years of education, APOE ε4, and BMI (see Table S2). Results of associations between identified metabolites/ratio and each cognitive performance domains are shown in Table S13. Correlations with global cognitive function were further validated in 92 individuals with both brain and serum samples and the directions were consistent with our previous findings among the entire cohort. Seven identified metabolites were all negatively correlated with global cognitive function in brain samples while two BAs showed negative correlations and five FFAs showed positive correlations in serum samples (Fig. S6). Additionally, the serum/brain ratio of identified FFAs were positively correlated with global cognitive function (i.e., lower levels of identified FFAs in serum and higher levels of identified FFAs in brain were associated with worse cognition) (Fig. S7).

### Identified metabolites predicted antecedent cognitive impairment before the manifestation of clinical symptoms

**Figure 3:**
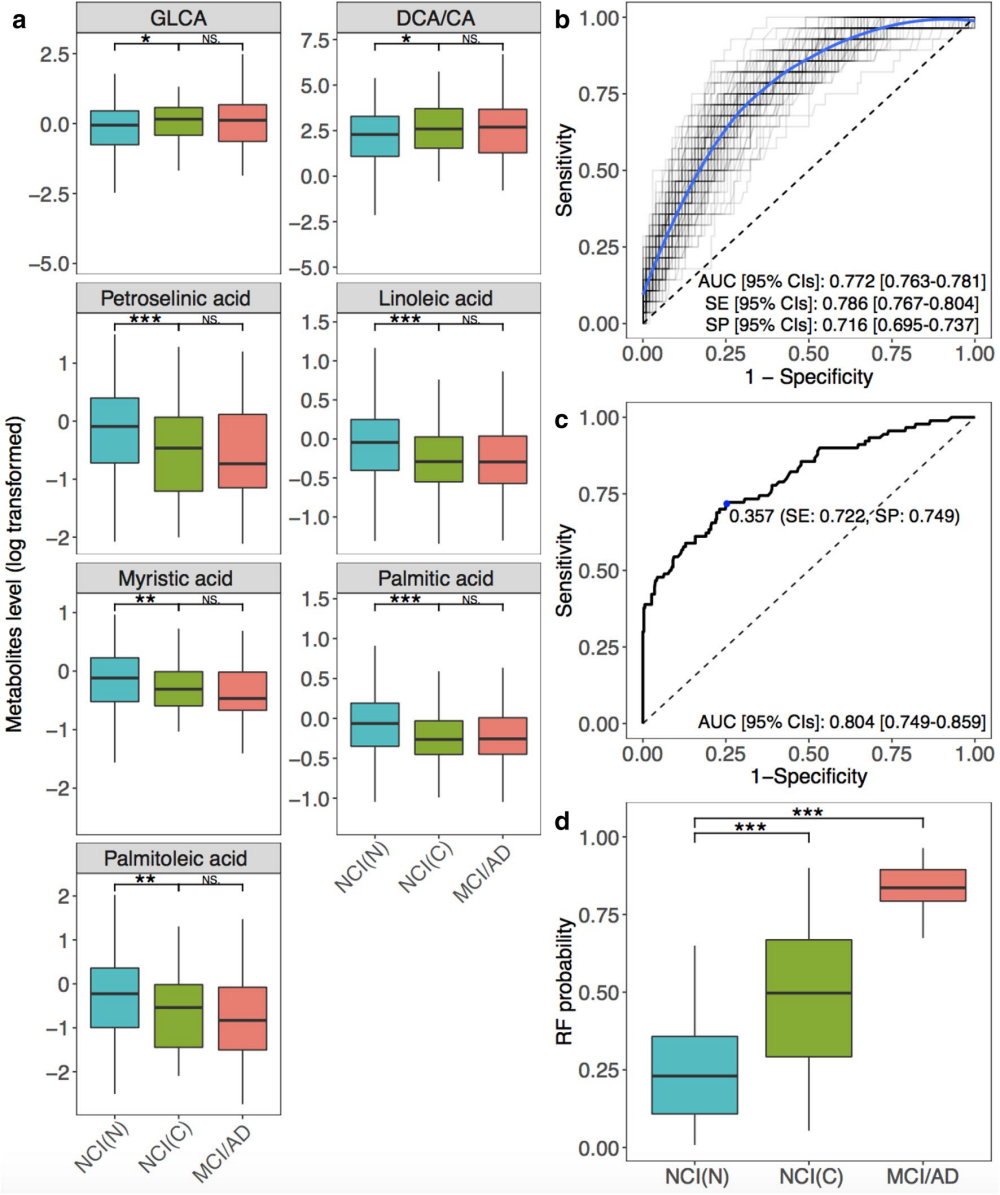
The identified panel of metabolites and its predictive performance. (**a**) Boxplots showing group differences and *P* values for identified metabolites across NCI (non-converters), NCI (converters), and MCI/AD for serum abundances. (**b**) ROC curves of metabolite models trained on the 70% training data and tested on the 30% testing data according to 100-times randomly training–testing splitting. (**c**) The ROC curve of the final metabolite model on the validation data. (**d**) RF scores of the final metabolite model across NCI (non-converters), NCI (converters), and MCI/AD. *P-value < 0.05, **P-value < 0.01, ***P-value < 0.001, Wilcoxon rank sum test. The optimal cutoff was determined by the Youden index. *AD* Alzheimer’s disease, *AUC* area under the receiver operating characteristic curve, *NCI(C)* NCI (converters), *NCI(N)* NCI (non-converters), *CIs* confidence intervals, *MCI* mild cognitive impairment, *NS* not significant, *SE* sensitivity, *SP* specificity.

The concentrations of GLCA and DCA/CA were significantly lower in the NCI (non-converters) group than in the NCI (converters) group. By contrast, the abundances of petroselinic acid, linoleic acid, myristic acid, palmitic acid, and palmitoleic acid were higher in the NCI (non-converters) group than in the NCI (converters) group (Fig. 3a). There were no significant differences in these metabolites between participants in NCI (converters) group vs. MCI/AD group (Fig. 3a). Using the seven metabolites and age, we built RF models on the 70% training set according to 100-times randomly splitting approach to differentiate MCI/AD patients from NCI (non-converters) group. The median of 100 times AUC on 30% testing set was 0.772 (95% CIs 0.763–0.781) with 0.786 SE (95% CIs 0.767–0.804) and 0.716 SP (95% CIs 0.695–0.737) using Youden's index to maximize the sum of SE and SP (Fig. 3b). RF models showed decent classification performances in differentiating MCI/AD group from NCI (non-converters).

Next, we were interested in studying the model’s early diagnostic capability for predicting NCI (converters) before clinical diagnosis. The model was thus applied on the entire NCI group at baseline to differentiate NCI (converters) from NCI (non-converters). We achieved an AUC of 0.804 (95% CIs 0.749–0.859, SE = 0.722, SP = 0.749 at the cutoff value of 0.357) (Fig. 3c) with significant differences in RF scores between NCI (converters) vs. NCI (non-converters), between NCI (non-converters) vs. MCI/AD group using the Wilcoxon rank-sum test (*P*-value < 0.001) (Fig. 3d). After additional adjustment for gender, years of education, APOE ε4, and BMI, fasting status, and medications (supplements, diabetes, lipid lowing), the RF scores remained significant (as an independent predictor) with a coefficient of 7.828 (*P*-value < 0.001) (Table S3). Additionally, the RF scores showed significant negative correlations with global cognitive function and the five cognitive domains with the same adjustment in mixed effects models (Table S12).

### Personalized metabolic pathway-based study for the association and prediction of cognitive impairment

**Figure 4:**
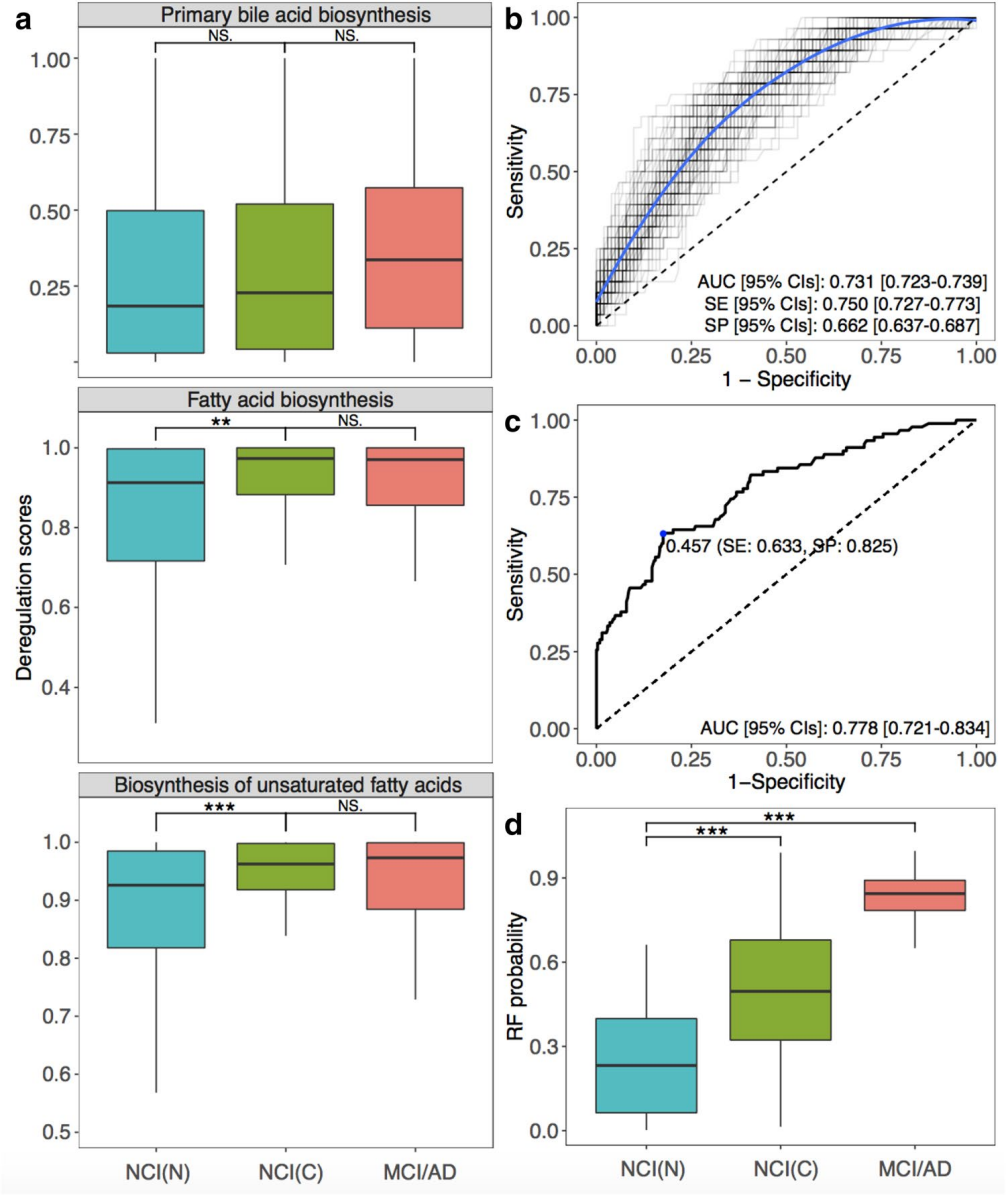
The pathway panel and its predictive performance. (**a**) Boxplots showing group differences and *P* values for identified pathways across NCI (non-converters), NCI (converters), and MCI/AD for serum abundances. (**b**) ROC curves of pathway models trained on the 70% training data and tested on the 30% testing data according to 100-times randomly training–testing splitting. (**c**) The ROC curve of the final pathway model on the validation data. (**d**) RF scores of the final pathway model across NCI (non-converters), NCI (converters), and MCI/AD. *P-value < 0.05, **P-value < 0.01, ***P-value < 0.001, Wilcoxon rank sum test. The optimal cutoff was determined by the Youden index. *AD* Alzheimer’s disease, *AUC* area under the receiver operating characteristic curve, *NCI(C)* NCI (converters), *NCI(N)* NCI (non-converters), *CIs* confidence intervals, *MCI* mild cognitive impairment, *NS* not significant, *SE* sensitivity, *SP* specificity.

Considering altered metabolite levels were significantly associated with cognitive impairment and showed early predictive value of clinical symptoms onset, we then employed the *pathifier* algorithm to summarize metabolite information to pathways level for further examinations. All PDS scores ranged from 0 to 1, where larger scores represent the higher extent of the abnormality in the corresponding metabolic pathway. 109 out of 177 metabolites detected in brain tissues and 102 out of 164 metabolites detected in sera were successfully mapped to the KEGG metabolic pathways. This method identified 52 metabolic pathways in brain tissues and 45 metabolic pathways in serum samples (44 overlapping pathways) (Figs. S1a,b, left panel; Table S9, Table S10), three of which (i.e., primary BAs biosynthesis, FFAs biosynthesis, and biosynthesis of unsaturated FFAs) were significantly shifted in both brain and serum samples (*P*-value < 0.05, and *Q*-value < 0.2, ordinal logistic regression for brain samples, logistic regression for serum samples) (Fig. S1a,b, right panel, Table S16, Table S17). We noted increased PDS for all three identified pathways from NCI to MCI/AD that suggested dysregulation of these metabolic pathways in MCI/AD patients compare to NCI. Detailed PDS of these pathways stratified by diagnostic groups are described in Table S4. Results also indicated that higher PDS were significantly associated with lower global cognitive function (i.e., worse cognitive performance) in both brain (ρ = − 0.16 for primary BAs biosynthesis pathway, ρ = − 0.23 for FFAs biosynthesis pathway, ρ = − 0.26 for biosynthesis of unsaturated FFAs pathway) (Fig. S4) and serum samples (ρ = − 0.16 for primary BAs biosynthesis pathway, ρ = − 0.13 for FFAs biosynthesis pathway, ρ = − 0.14 for biosynthesis of unsaturated FFAs pathway) (Fig. S5 respectively. In Table S5, we show the significant negative associations between each cognitive test and PDS of three pathways after adjusting for age, gender, years of education, and APOE ε4 (additional adjustment for BMI in serum samples). Two fatty acid pathways showed significantly different PDS between the NCI (non-converters) group and the NCI (converters) group (*P*-value = 0.0012, and *P*-value < 0.001), respectively. A gradually increasing trend was noted for the BAs pathway across groups (i.e., NCI (non-converters) < NCI (converters) and MCI/AD) (Fig. 4a).

We then constructed a discriminant RF model in 70% training data and tested on 30 testing data based on three identified metabolic pathways along with age to differentiate MCI/AD from NCI (non-converters) in model construction data using 100-times randomly splitting approach. The median AUC on the testing set was 0.731 (95% CIs = 0.723–0.739) with 0.750 SE (95% CIs = 0.727–0.773) and 0.662 SP (95% CIs = 0.637–0.687) (Fig. 4b). Applying the RF model to the whole NCI data at baseline could successfully discriminate NCI (converters) from NCI (non-converters) with an AUC of 0.778 (95% CIs = 0.721–0.834), SE = 0.633, SP = 0.825, cutoff value = 0.457 (Fig. 4c). Similarly, predictive RF scores were significantly different between NCI (converters) vs. NCI (non-converters), and NCI (non-converters) vs. MCI/AD group (*P*-value < 0.001) (Fig. 4d). After adjusting for gender, years of education, APOE ε4, BMI, fasting status, and medications (supplement, diabetes, lipid lowing), the RF scores remained statistically significant as an independent predictor with a coefficient of 5.629 (*P*-value < 0.001) (Table S6).

### Identified metabolites and metabolic pathway were associated with neuropathology

Notably, we observed significant differences (gradually increasing trend) of metabolite abundances in brain tissue across Braak scores: DCA/CA ratio (*P*-value = 0.021), petroselinic acid (*P*-value = 0.038), linoleic acid (*P*-value = 0.041), palmitic acid (*P*-value = 0.043), and palmitoleic acid (*P*-value = 0.038) (Fig. S8a). Consistently, the abundance of six metabolites in brain were higher in the CERAD AD group (score 1 ~ 2) than the CERAD non-AD group (score 3 ~ 4): GLCA (*P*-value = 0.041), petroselinic acid (*P*-value = 0.014), linoleic acid (*P*-value < 0.001), myristic acid (*P*-value = 0.015), palmitic acid (*P*-value = 0.026), and palmitoleic acid (*P*-value = 0.0019) (Fig. S8b). Brain PDS of identified pathways were gradually increased across Braak scores, where the biosynthesis of unsaturated FFAs pathway differed with statistical significance (*P*-value = 0.036) (Fig. S2). Higher brain PDS were associated with a significantly greater risk of neuritic plaque burden based on CERAD criteria: primary BAs biosynthesis (*P*-value = 0.04), FFAs biosynthesis (*P*-value = 0.028), and biosynthesis of unsaturated FFAs (*P*-value = 0.0089) (Fig. S3).

## Discussion

The alterations of metabolic profiles, especially in the brain, of MCI and AD patients have not been extensively studied. In our study, we comprehensively measured 164 and 177 metabolites in serum and brain samples, respectively. Our findings showed six significantly differential metabolites and one ratio in both brain and serum samples (petroselinic acid, linoleic acid, myristic acid, palmitic acid, and palmitoleic acid, GLCA, and DCA/CA ratio). We observed the differences of serum metabolites were not as strong as those in the brain samples which is reasonable considering peripheral circulation serves as a down-stream pool of all the pathological perturbations, and thus could be noisier than brain samples. However, in the following analysis, the identified metabolites/ratios showed consistent associations with neuropathology results, and cognitive function regardless of adjustments for potential confounding factors (i.e., age, gender, years of education, APOE ε4, BMI, and medications). AD is a progressive neurodegenerative disease with neuropathological changes commonly observed. Although brain tissues used in our research were obtained and implemented upon death, studies have shown that there is a strong independent correlation between autopsy neuropathological hallmarks with cognitive impairment severity^51^. Thus, we believe both neuropathology results and cognitive function tests are AD markers which were correlated with identified metabolites in our study. Additionally, these metabolites demonstrated group differences of NCI (converter) vs. NCI (non-converter) within baseline normal controls which indicated their potential predictive value. A personalized pathway-level analysis further demonstrated our findings of FFAs and BAs metabolic perturbations among cognitively impaired persons and the predictive value of using personalized pathway information.

Altered FAs profiles were found in different regions of brain tissue in AD patients and linked to neuropathology and cognitive performance^14^. Our study showed five significant increments of brain FFAs, while decrements of these FFAs appeared in the serum of AD patients. The serum/brain ratio of identified FFAs were positively correlated with global cognitive function (i.e., lower levels of identified FFAs in serum and higher levels of identified FFAs in brain were associated with worse cognition) (Fig. S7).

**Figure 5:**
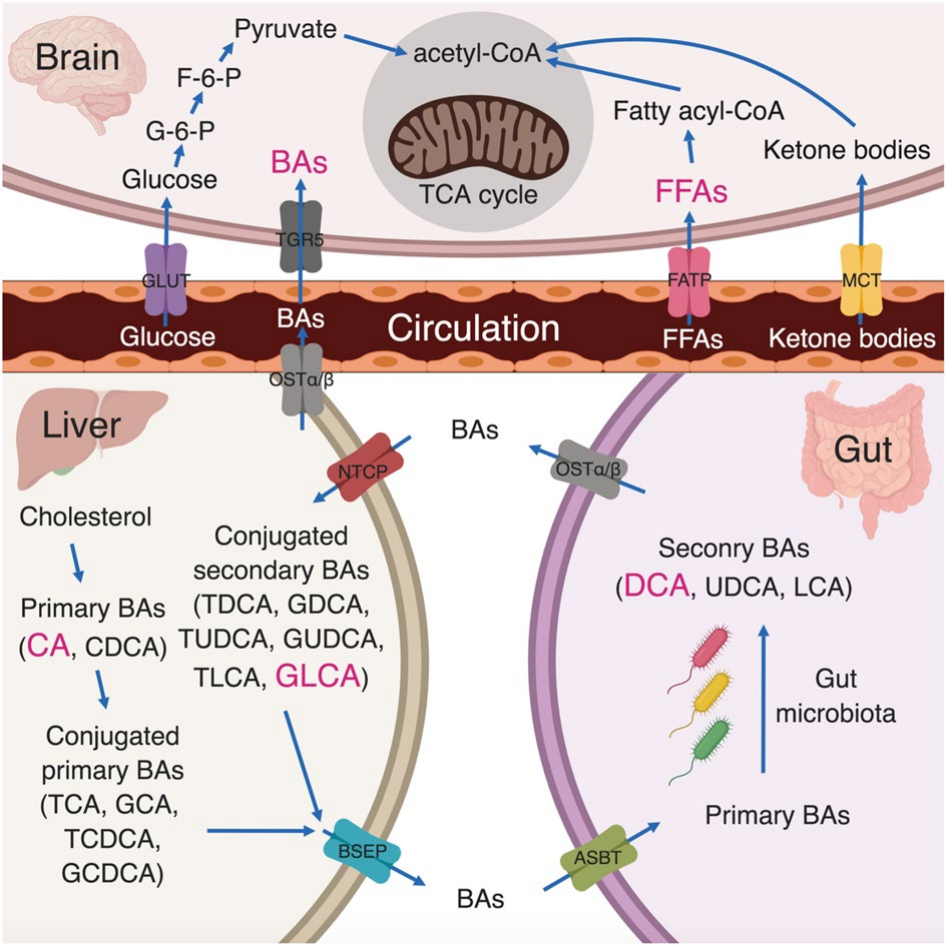
Pathways involved in FFAs and BAs. Healthy neurons are highly glycolytic, catabolizing rate of glucose consumption through the glycolysis and TCA cycle to produce ATP. Reduced glucose utilization and metabolic dysfunction could be detected using FDG-PET and metabolomics approaches in AD patients. The metabolic instability with decreased glucose utilization in impaired neurons can cause the energy supply shift towards alternative energy sources, e.g., FFAs and ketone bodies. Primary BAs are synthesized in the liver from cholesterol. A dysfunction of the gut microbiome can cause the accumulation of cytotoxic secondary bile acids, e.g., DCA and GLCA, which can be secreted into the systemic circulation and then across the blood–brain barrier to enter the brain. *ASBT* apical sodium-dependent bile acid transporter, *BSEP* bile salt export pump, *CA* cholate, *CDCA* chenodeoxycholate, *CoA* coenzyme A, *DCA* deoxycholate, *F-6-P* fructose-6-phosphate, *FATP* fatty acid transporter, *G-6-P* glucose-6-phosphate, *GCA* glycocholate, *GCDC*A glycochenodeoxycholate, *GDCA* glycodeoxycholate, *GLCA* glycolithocholate, *GLUT* glucose transporter, *GUDCA* glycoursodeoxycholate, *LCA* lithocholate, *MCT* monocarboxylate transporter, *NTCP* sodium/taurocholate co-transporting polypeptide, *OST* organic solute and steroid transporter, *TCA* tricarboxylic acid, *TCA* taurocholate, *TCDCA* taurochenodeoxycholate, *TDCA* taurodeoxycholate, *TLCA* taurolithocholate, *TUDCA* tauroursodeoxycholate, *TGR5* G protein–coupled bile acid receptor, *UDCA* ursodeoxycholate.

Our findings are consistent with prior observations in AD mice that the significant accumulation of FFAs in the hippocampus and cortex, including palmitoleic acid, palmitic acid, and linoleic acid, might be associated with the utilization of free fatty acid in the brain^26^. It is well-known that the brain is one of the most energy-demanding organs with a high glycolytic, catabolizing rate of glucose consumption. The energy supply shift from glucose towards alternative energy sources, (e.g., ketone bodies and FFAs) has been observed in other neurological disorders including schizophrenia^52^ (Fig. 5). More interestingly, at the early stages of AD, reduced glucose utilization and metabolic dysfunction can be detected using FDG-PET, which is one of the earliest detectable symptoms of AD^53^. The metabolic instability with decreased glucose utilization in impaired neurons among AD patients occurs up to twenty years prior to the onset of clinical symptoms indicating that metabolic decline may contribute to the development of cognitive impairment^54^. Previous studies suggested that fatty acids can across the blood–brain barrier (BBB) via simple diffusion. At the same time, fatty acid transport proteins are also involved in this process. Fatty acid binding protein 5 (FABP-5), fatty acid transport proteins-1 (FATP-1), FATP-4, and fatty acid translocase (CD36) are the key FFA transport proteins and these transporters are expressed in human brain microvessel endothelial cells (HBMEC)^55^. The significant decrement of the movement for many FFAs (including linoleic acid, myristic acid, and palmitic acid) from apical medium to the basolateral medium across HBMEC monolayer with the knockdown of these FFA transport proteins was observed^55^. Additionally, many studies demonstrated that the CD36 gene is associated with AD^56,57^, and the BBB damage may also facilitate the transferring of FFAs from blood to brain in AD patients^58^.

In addition to the FFAs dysregulation, the association between cholesterol metabolism and AD leads to substantial research interests of how BAs profile changes in cognitively impaired and AD patients^59^. Furthermore, BAs play a major role in regulating energy homeostasis through binding to nuclear receptors and evidence has shown that both primary and secondary BAs could cross the BBB via a gut-liver-brain axis^60,61^. In this study, we observed one secondary conjugated BA (i.e., GLCA) and one primary-secondary BAs ratio (DCA/CA) increased in both serum and brain tissue in MCI/AD patients compared to NCI, which was consistent with previous findings^12^. Primary BAs are synthesized in the liver from cholesterol and are then bio-transformed into secondary BAs by the gut microbiome (Fig. 5). The increasing DCA/CA ratio seen here might suggest a dysfunction of bacterial 7α-dehydroxylases which leads to the accumulation of cytotoxic secondary BAs. Similarly, GLCA, another cytotoxic BA, was increased in our study. The survival analysis conducted by Mahmoudian Dehkordi et al*.* on MCI conversion to AD showed significantly changed prognostic endpoints when splitting samples into different groups according to BA levels^12^, which also suggested that the total BAs/BA ratios could serve as potential predictors for the conversion of ADs among non-AD clinical groups.

Human CSF-based studies also detected metabolic changes between controls and MCI and AD patients. One study demonstrated that altered fatty acids levels in CSF reflected the importance of abnormal metabolism in AD and suggested that disturbed fatty acids metabolism might contribute to AD pathology^62^. Similarly, Trushina et al.^63^ found the BA pathway was significantly altered in both plasma and CSF of AD vs. NCI subjects and significant alterations in fatty acid metabolism in the plasma of MCI patients and in the CSF of AD patients.

While this study was conducted on a well-characterized longitudinal cohort it is not without its limitations. First, the sample size of brain tissues in this study is relatively limited due to the study design which required available ante-mortem MRI. Additional work with a larger number of brain tissues is ongoing. In addition, there are only 11 AD subjects with serum samples. We expect further validating our finding in more serum samples with an ordinal group level (NCI → MCI → AD), as was done with brain samples as more data becomes available. Second, the current data is cross-sectional preventing us from exploring serum metabolome changes over time. More analyses can be performed to further explore the metabolite changes in the development of AD over time. Third, although we validated our prediction models on the entire NCI group to show its additional predictive values in differentiating NCI (converters) from NCI (non-converters), more external data are needed for validation. Fourth, in this study, we utilized GC-TOFMS and LC-TQMS platforms to quantitatively measure 177 metabolites in brain samples and 164 in serum samples (mainly amino acids, bile acids, carbon hydrates, organic acids, and fatty acids). It would be interesting to measure broader untargeted metabolic profiles and more metabolites of AD serum and brain samples in the future studies. Fifth, our findings suggest the emerging role for gut microbiome in the development of AD. Thus, further experimental microbiota studies could provide a better understand of how the gut-brain axis plays a role in AD development. Last, since our results provide a proof-of-concept for the feasibility of early detection among NCI subjects at high risk of developing cognitive impairment, future clinical studies can be designed to explore the benefits of early interventions. The study also has strengths in that the follow-up and autopsy rates in the parent cohorts are very high leading to excellent internal validity. The diagnostic groups were comparable as they came from a single larger cohort.

Collectively, our findings present a new point-of-view into the pre-clinical evolution of AD and lend strength to the hypothesis that individuals with higher risks of cognitive impairment can be identified before the development of overt symptoms via a metabolomics approach. To this end, we provide two predictive models: one based on differentially expressed metabolites and the other on identified metabolic pathways. Varma et al*.* utilized machine learning approaches to identify potential metabolites related to AD pathology and progression^18^. We took their work one step further by constructing machine learning models to discriminant the final-state healthy controls vs. MCI/AD patients. The 100 times resampling results demonstrated the feasibility and robustness of our predictive models. Particularly, by employing our models on the baseline group, we successfully identified the high-risk subgroup (i.e., NCI (converters)) several years before the clinical diagnosis.

## Supplementary information

Supplementary Information.

## Data Availability

Metabolomics datasets used in the current analyses for the ROS/MAP cohorts are available via the Accelerating Medicines Partnership-Alzheimer’s Disease (AMP-AD) Knowledge Portal and can be accessed at 10.7303/syn10235594. The full complement of clinical and demographic data for the ROS/MAP cohorts are available via the Rush AD Center Resource Sharing Hub and can be requested at https://www.radc.rush.edu.
