# Supplementary material for: Peripheral serum metabolomic profiles inform central cognitive impairment
Source: Sci Rep. 2020 Aug 20;10:14059. doi: 10.1038/s41598-020-70703-w (PMC7441317; doi:10.1038/s41598-020-70703-w)
Supplement: Supplementary file 1 — Supplementary Information. [file 41598_2020_70703_MOESM1_ESM.pdf]

Supporting Information

Peripheral Serum Metabolomic Profiles Inform Central Cognitive Impairment

Jingye Wang, Runmin Wei, Guoxiang Xie, Matthias Arnold, Alexandra Kueider-Paisley, Gregory Louie, Siamak MahmoudianDehkordi, Colette Blach, Rebecca Baillie, Xianlin Han, Philip L. De Jager, David A. Bennett, Rima Kaddurah-Daouk, Wei Jia

Checklist of Supporting Information

Figure S1. Brain metabolic pathways and serum metabolic pathways composition and alterations

Figure S2. PDS of metabolic pathways across Braak scores in brain

Figure S3. PDS of metabolic pathways across CERAD scores in brain

Figure S4. Correlations between PDS of metabolic pathways and global cognitive function in brain

Figure S5. Correlations between PDS of metabolic pathways and global cognitive function in sera

Figure S6. Associations between metabolites level and global cognitive function among participants with both brain and serum samples.

Figure S7. Associations between level of serum/brain FFAs ratios and global cognitive function among participants with both brain and serum samples.

Figure S8. Associations between metabolites level and Braak scores, CERAD scores.

Figure S9. The workflow chart of the data and the analysis.

Figure S10. Histogram of Time Difference among NCI subjects' serum samples

Supplementary Text.

Table S1. List of cognitive performance tests

Table S2. Associations between identified metabolites/ratio and cognitive performance tests adjusting for age, gender, years of education, APOE ε4, BMI

Table S3. Logistic regression of metabolite marker panel-based RF score to discriminate NCI (converters) vs. NCI (non-converters) adjusting for gender, years of education, APOE ε4, and BMI

Table S4. PDS of metabolic pathways differentially expressed in participants with cognitive decline

Table S5. Associations between identified metabolic pathways and cognitive performance tests adjusting for age, gender, years of education, APOE ε4, and BMI

Table S6. Logistic regression of metabolic pathway panel-based RF score to discriminate NCI (converters) vs. NCI (non-converters) adjusting for gender, years of education, APOE ε4, and BMI

Table S7. Levels of detected metabolites across clinical groups in brain samples

Table S8. Levels of detected metabolites across clinical groups in serum samples

Table S9. PDS of mapped metabolic pathways across clinical groups in brain samples

Table S10. PDS of detected metabolic pathways across clinical groups in serum samples

Table S11. Levels of identified metabolites in samples with both brain and blood metabolomics data

Table S12. Mixed effects model of metabolite marker panel-based RF score adjusting for age, gender, years of education, APOE ε4, and BMI

Table S13. Associations between identified metabolites/ratio and cognitive performance domains adjusting for age, gender, years of education, APOE ε4, and BMI.

Table S14. P values and Q values of identified metabolites across clinical groups in brain samples using ordinal regression.

Table S15. P values and Q values of identified metabolites across clinical groups in serum samples using logistic regression.

Table S16. P values and Q values of identified pathway across clinical groups in brain samples using ordinal regression.

Table S17. P values and Q values of identified pathway across clinical groups in serum samples using logistic regression.

Table S18. P values identified pathway across clinical groups in serum samples adjusting for other potential confounders using logistic regression.

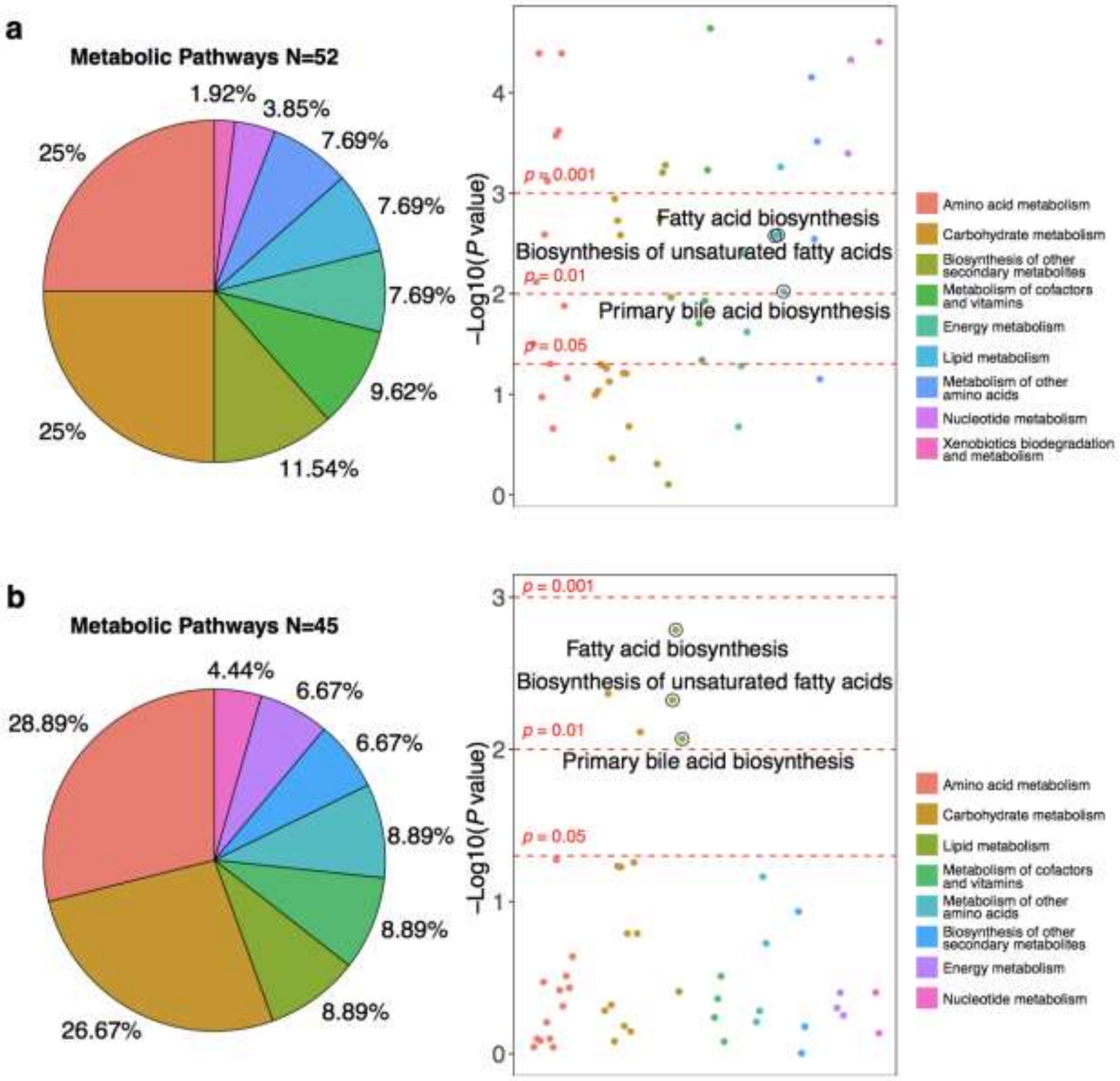

Figure S1. Brain metabolic pathways and serum metabolic pathway compositions and alterations.

(a) Left panel: the mapped pathway composition in brain. Right panel: –log10 (*P*-value) across clinical groups (NCI, MCI, AD). (b) Left panel: the mapped pathways composition in serum. Right panel: –log10 (*P*-value) across clinical groups (NCI, MCI/AD). Abbreviations: AD – Alzheimer’s disease; MCI – mild cognitive impairment; NCI: no cognitive impairment

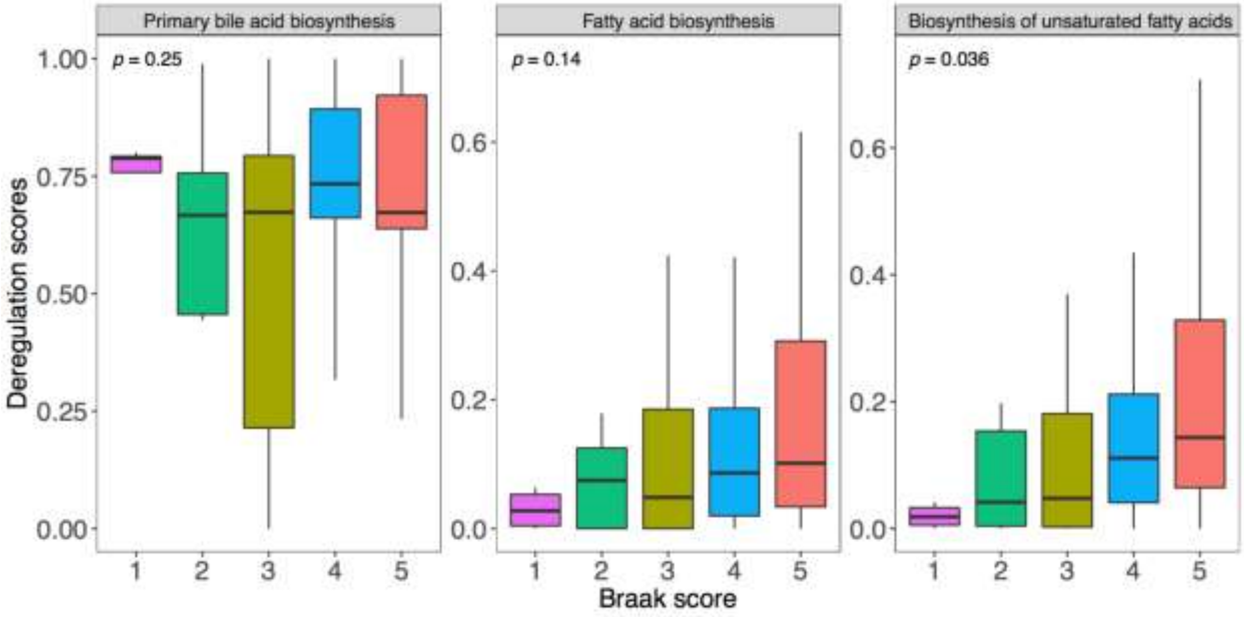

Figure S2. PDS of metabolic pathways across Braak scores in brain.

Boxplots showing group differences and *P*-values for identified pathways across Braak groups for brain tissues.

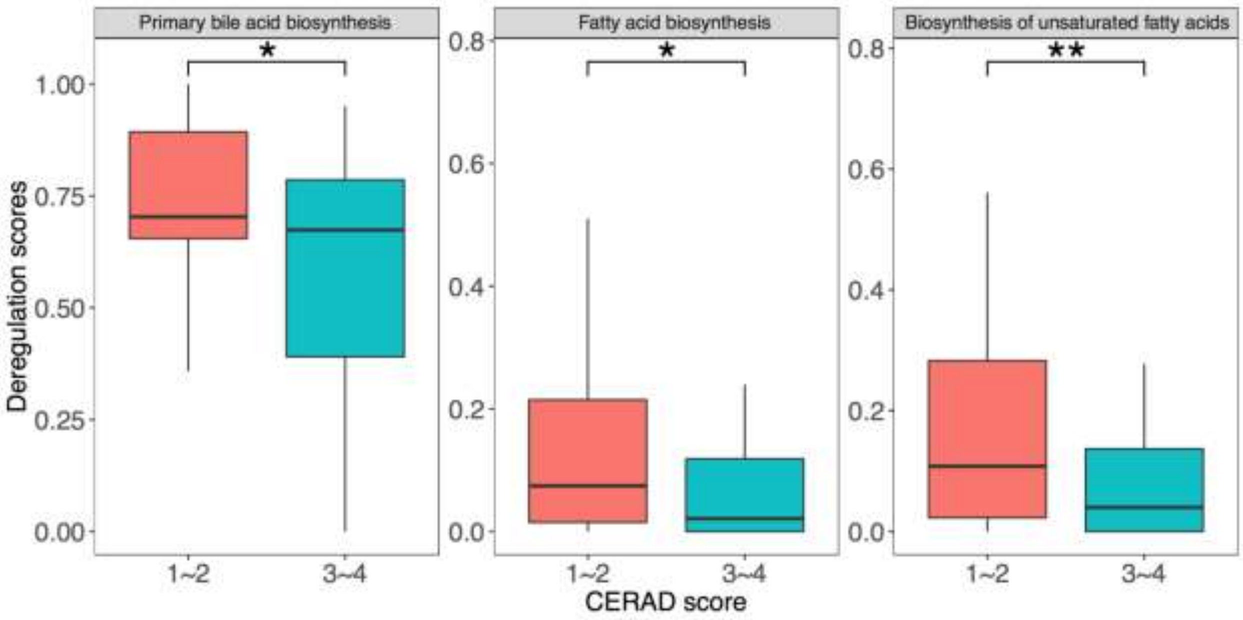

Figure S3. PDS of metabolic pathways across CERAD scores in brain.

Boxplots showing group differences and significances for identified pathways across CERAD groups for brain tissues. * *P*-value < 0.05, ** *P*-value < 0.01, *** *P*-value < 0.001, Wilcoxon rank sum test. CERAD, Consortium to Establish a Registry for Alzheimer's Disease

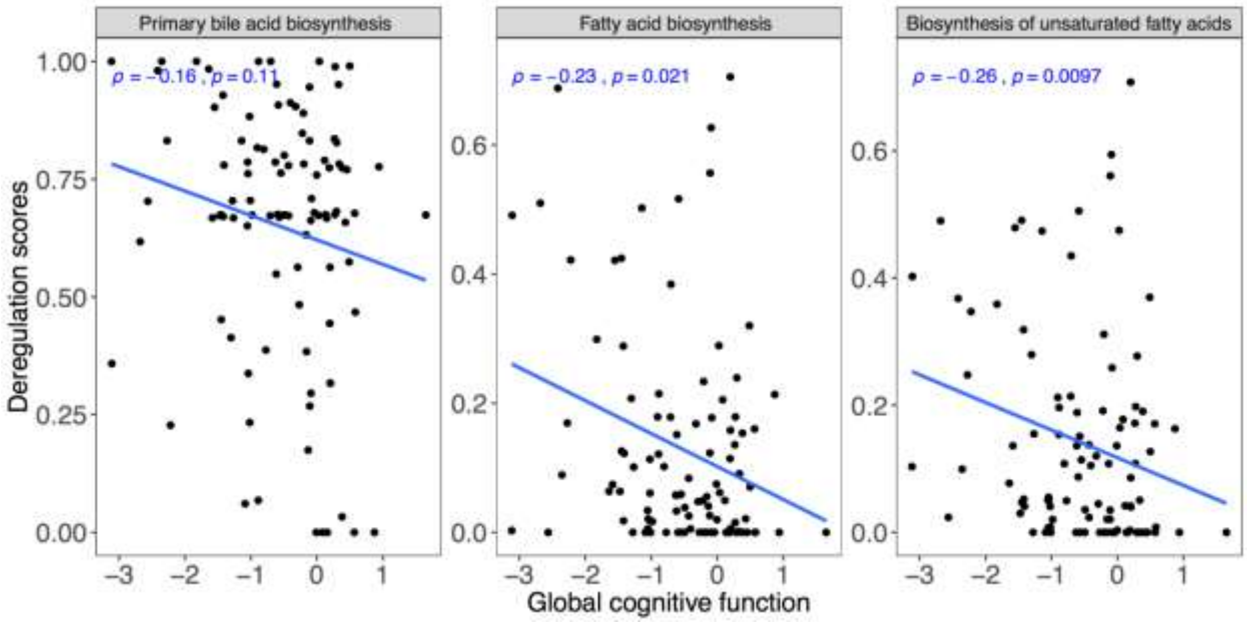

Figure S4. Correlations between PDS of metabolic pathways and global cognitive function in brain.

Scatterplots with ρs and *P*-values showing correlations between brain pathway PDS and global cognitive function. ρ, correlation coefficient of Spearman's rank correlation test.

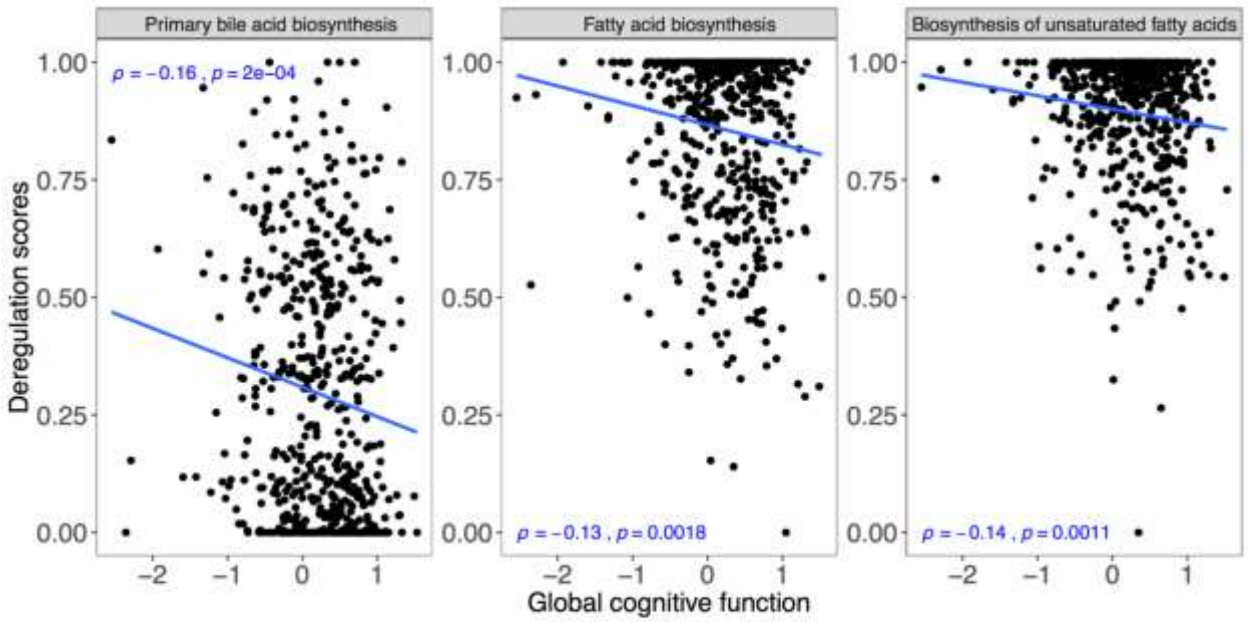

Figure S5. Correlations between PDS of metabolic pathways and global cognitive function in sera.

Scatterplots with ρs and *P*-values showing correlations between serum pathway PDS and global cognitive function. ρ, correlation coefficient of Spearman's rank correlation test.

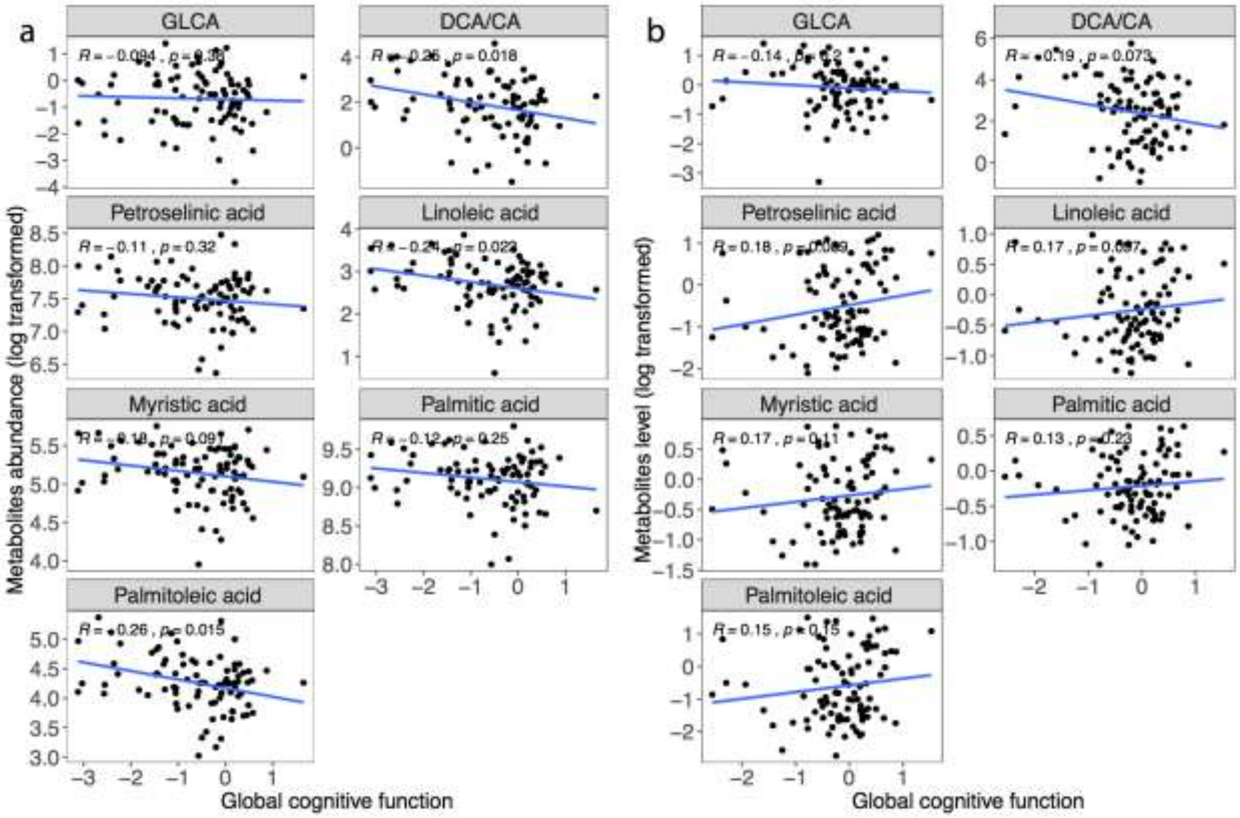

Figure S6. Associations between metabolites level and global cognitive function among participants with both brain and serum samples.

(a) Scatterplots with ρs and p-values showing associations between brain metabolites abundances and global cognitive function. (b) Scatterplots with ρs and p-values showing associations between serum metabolites abundances and global cognitive function. ρ, correlation coefficient of Spearman's rank correlation test.

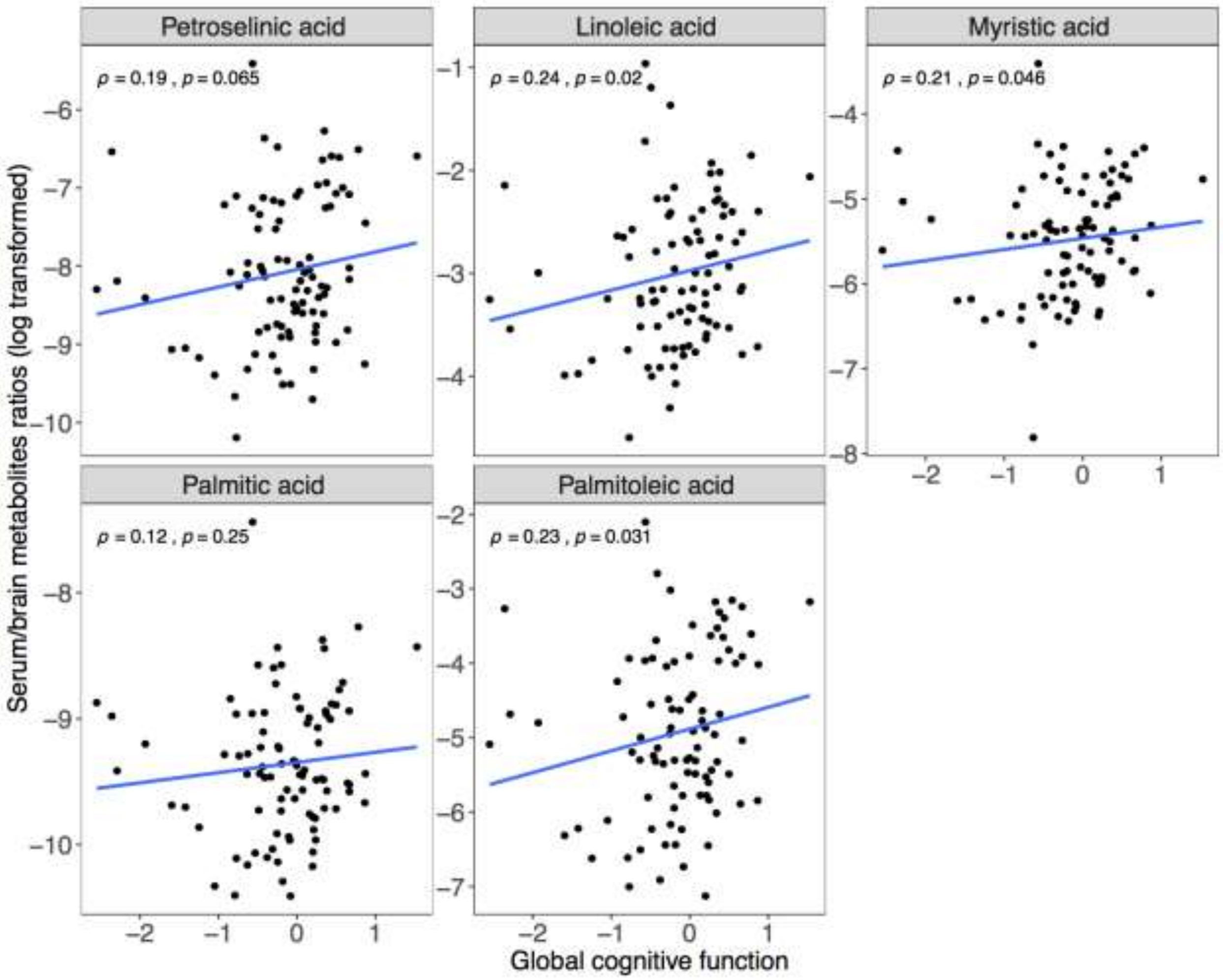

Figure S7. Associations between level of serum/brain FFAs ratios and global cognitive function among participants with both brain and serum samples.

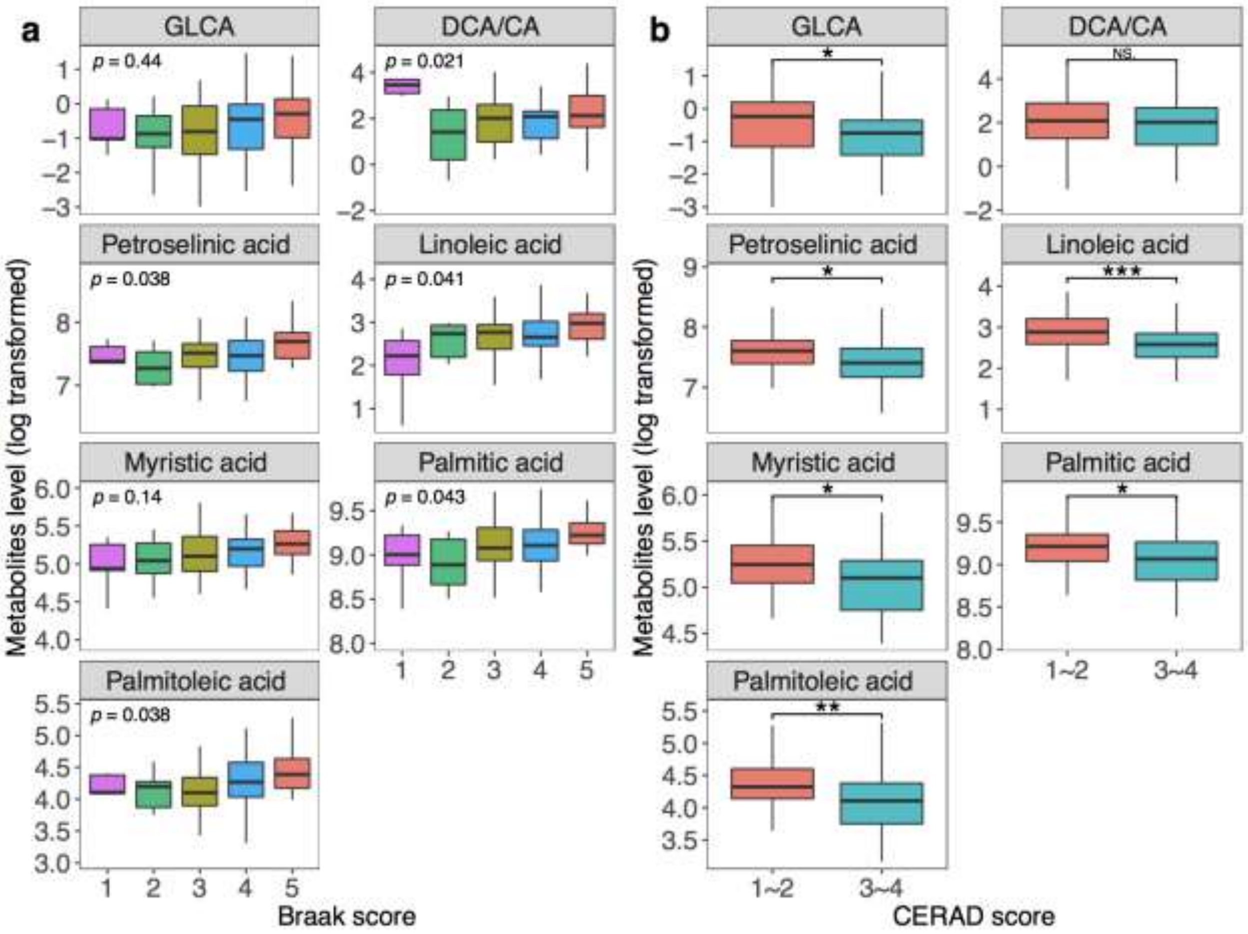

Figure S8. Associations between metabolites level and Braak scores, CERAD scores.

(a) Boxplots showing group differences and *P* values for identified metabolites across Braak groups for brain tissue abundances. (b) Boxplots showing group differences and significances for identified metabolites across CERAD groups for brain tissue abundances.

* *P*-value < 0.05, ** *P*-value < 0.01, *** *P*-value < 0.001, Wilcoxon rank sum test.

CERAD, Consortium to Establish a Registry for Alzheimer's Disease; NS, not significant.

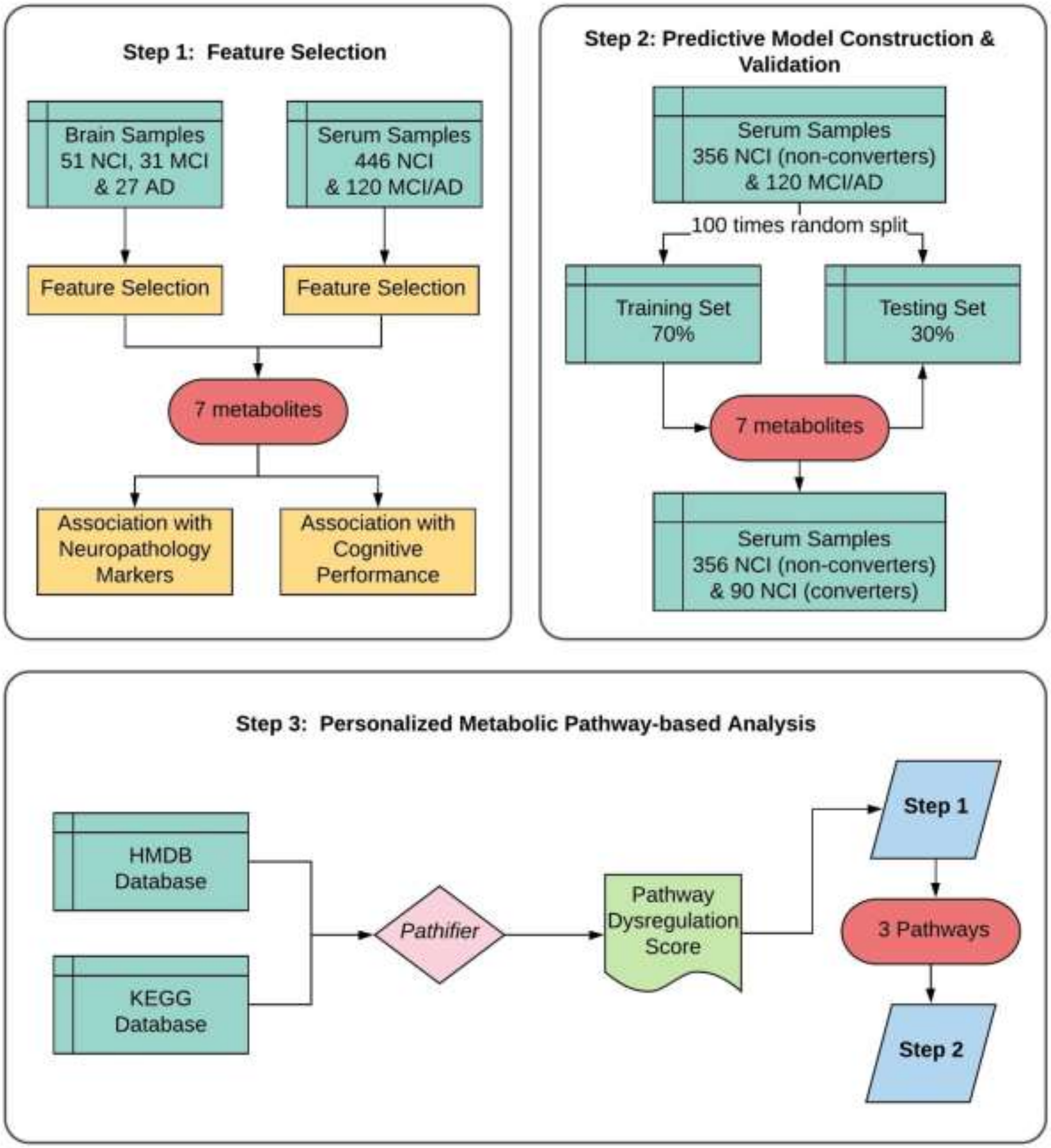

Figure S9. The workflow chart of the data and the analysis.

Supplementary Text

Serum sample preparation

We mixed an aliquot of 50 μL serum with 150 μL methanol, and vortexed the mixture for 2 min, let it stand for 10 min and centrifuged at 4 °C for 10 min. We then transferred 160 μL of the supernatant to a clean tube and vacuum dried the remaining aliquot re-dissolved it with a matched amount of acetonitrile (0.1% formic acid) and added water (0.1% formic acid) to a volume of 40 μL. The supernatant after the centrifugation was used for UPLC-TQMS and GC-TOFMS analysis. A mixture of 20 μL from the final supernatant of each sample was prepared for use as pooled quality control (QC) samples.

Brain sample preparation

We weighted 30 mg brain tissue, homogenized it with 75 μL of 50% precooled methanol using a Bullet Blender Tissue Homogenizer (Next Advance, Inc., Averill Park, NY) for 3 min. After centrifugation at 4 °C for 15 min, we transferred the supernatant to a clean tube. We performed the second step extraction by adding precooled methanol and chloroform mixture (3:1) to the residue followed by centrifugation. We combined the supernatant with the previous one and vortexed the mixture for 5 min and performed centrifugation for 15 min. The supernatant was used for UPLC-TQMS and GC-TOFMS analysis. A mixture of 20 μL from the final supernatant of each sample was prepared for use as pooled QC samples.

Quality control Procedure

Previously prepared QC samples were run between every ten sample injections. For each metabolite in QC samples, we calculated the relative standard deviations (RSDs), which was less than 15% for batches of samples.

Table S1. List of cognitive performance tests.

**Episodic memory**	
cts_wli	word list
cts_wlii	word list recall
cts_wliii	word list recognition
cts_ebmt	East Boston immediate recall
cts_ebdr	East Boston delayed recall
cts_story	Logical memory I (immediate recall)
cts_delay	Logical memory II (delayed recall)
**Working memory**	
cts_df	digits forward
cts_db	digits backward
cts_doperf	digit ordering
**Semantic memory**	
cts_catflu	category fluency (animals, fruits)
cts_bname	Boston naming
cts_read_nart	reading test
**Perceptual orientation**	
cts_lopair	line orientation
cts_pmat	progressive matrices
**Perceptual speed**	
cts_sdmt	symbol digits modality test
cts_nccrtd	number comparison
cts_stroop_cname	stroop color naming
cts_stroop_wread	stroop word reading
**Individual cognitive performance test scores**	
cts_mmse30	Mini-Mental State Exam

Table S2. Associations between identified metabolites/ratio and cognitive performance tests adjusting for age, gender, years of education, APOE ε4, and BMI.

	GLCA			DCA/CA			Petroselinic acid			Linoleic acid			Myristic acid			Palmitic acid			Palmitoleic acid		
	Estimate	SE	P value	Estimate	SE	P value	Estimate	SE	P value	Estimate	SE	P value	Estimate	SE	P value	Estimate	SE	P value	Estimate	SE	P value
**Brain samples**																					
Episodic memory																					
CERAD word list	-0.4379	0.60532	0.47139	-0.94862	0.47727	0.05004	-3.7828	1.60013	0.02033*	-1.5494	1.16385	0.18662	-2.33797	1.76966	0.18996	-5.158	1.86831	0.00705**	-4.2995	1.352	0.00205**
CERAD word list recall	-0.38801	0.28455	0.17626	-0.65116	0.2203	0.00402**	-2.16152	0.7467	0.00481**	-0.77084	0.55074	0.16521	-1.49384	0.83129	0.07584	-2.83288	0.87142	0.00164**	-1.8495	0.647	0.00534**
East Boston-immediate recall	-0.06896	0.30093	0.81925	-0.35067	0.24184	0.1505	-2.02802	0.83262	0.01681*	-1.2666	0.57071	0.02895*	-2.40666	0.86467	0.00654**	-2.25246	0.92829	0.01722*	-2.4051	0.6617	0.00046***
East Boston-delayed recall	-0.03223	0.39918	0.93582	-0.82352	0.31404	0.01026*	-2.68397	1.11155	0.01778*	-1.97045	0.74884	0.01001*	-2.94574	1.15962	0.01279*	-3.35974	1.22298	0.00726**	-3.0923	0.8977	0.00087***
Logical Memory I - immediate	0.17366	0.62868	0.78304	-0.74019	0.49482	0.13839	-3.54226	1.77609	0.04931*	-1.97905	1.25197	0.11765	-1.8854	1.89217	0.32187	-4.3939	1.98874	0.02984*	-3.3394	1.4932	0.02794*
Logical Memory II - delayed	-0.56911	0.64921	0.38325	-0.69569	0.52361	0.18765	-3.51332	1.84168	0.05993	-1.1477	1.30483	0.38166	-1.93393	1.96515	0.32796	-4.84871	2.02898	0.01915*	-3.2021	1.5536	0.04247*
Working memory																					
Digit ordering	0.01081	0.16564	0.94813	-0.30739	0.13368	0.02379*	-0.9832	0.44324	0.02906*	-0.55361	0.31546	0.08267	-1.11305	0.47957	0.02255*	-1.15903	0.51887	0.02798*	-1.0564	0.3808	0.00673**
Semantic memory																					
Category fluency (animals + fruits)	-0.47043	1.2602	0.70977	-1.58069	1.0032	0.11846	-7.28133	3.32774	0.03114*	-4.25996	2.36124	0.07442	-9.45028	3.55827	0.00929**	-10.9194	3.86668	0.00579**	-7.8141	2.7816	0.00604**
Category fluency - animals	-0.10997	0.65268	0.86656	-0.66865	0.52154	0.20298	-4.22325	1.71124	0.0154*	-2.35554	1.21922	0.05637	-5.4575	1.82482	0.00355**	-6.34297	1.9793	0.00185**	-4.398	1.4288	0.00273**
Category fluency - fruits	-0.33544	0.6691	0.61732	-0.87011	0.53659	0.10829	-3.20303	1.78613	0.07618	-1.8939	1.25936	0.136	-4.20837	1.92007	0.03089*	-4.62034	2.08337	0.02901*	-3.5464	1.4979	0.01998*
Boston naming	0.13813	0.2495	0.58133	-0.44798	0.19275	0.0226*	-0.6253	0.66814	0.35208	-0.77816	0.47194	0.10301	-0.28762	0.72655	0.69323	-0.81311	0.786	0.30395	-0.6747	0.5847	0.25184

Perceptual speed																					
Symbol digit modality	-1.44246	1.39571	0.30461	-2.48162	1.10378	0.02742*	-3.80028	3.73003	0.31147	-3.81862	2.58628	0.14389	-7.77863	3.96174	0.05321	-6.78738	4.29413	0.11806	-5.1037	3.1556	0.10989
Individual cognitive performance test scores																					
MMSE	-0.01418	0.6511	0.98266	-1.74918	0.48574	0.00051***	-2.89725	1.70631	0.09279	-2.55611	1.21308	0.03774*	-4.00233	1.84761	0.0328*	-3.61047	2.04151	0.08018	-4.3431	1.3915	0.00239**
**Serum samples**																					
Episodic memory																					
CERAD word list	-0.45512	0.24035	0.05892	-0.2346	0.1455	0.10759	0.94355	0.3022	0.00191**	1.12047	0.48225	0.02061*	1.14766	0.47546	0.01619*	0.94256	0.49143	0.05576	0.76647	0.2605	0.00343**
CERAD word list recall	-0.2345	0.12238	0.05597	-0.22181	0.07348	0.00268**	0.35284	0.15447	0.02283*	0.30313	0.24662	0.21967	0.26855	0.24328	0.27024	0.11843	0.25079	0.63701	0.32623	0.1329	0.01447*
Working memory																					
Digit forward	-0.00976	0.09474	0.91803	-0.08073	0.05704	0.1577	0.18244	0.11907	0.12618	0.33794	0.18937	0.07501	0.31732	0.18681	0.0901	0.0943	0.19298	0.62534	0.20349	0.1024	0.04755*
Digit ordering	0.12316	0.07593	0.10551	-0.11911	0.04587	0.00972**	0.13732	0.09669	0.15627	0.18593	0.15349	0.22643	0.23285	0.15115	0.12416	0.08285	0.15626	0.59624	0.20811	0.0828	0.01235*
Semantic memory																					
Category fluency (animals + fruits)	-0.24002	0.45942	0.60162	-0.69523	0.27539	0.01193*	1.7249	0.57597	0.0029**	2.3403	0.91884	0.0112*	2.48348	0.90491	0.00631**	1.19175	0.93815	0.20464	1.44255	0.4963	0.00383**
Category fluency - animals	-0.21257	0.24867	0.39309	-0.34237	0.14932	0.02232*	0.84164	0.31251	0.00735**	1.17678	0.4981	0.01858*	1.07823	0.49154	0.02878*	0.5968	0.5082	0.24089	0.60626	0.2698	0.02511*
Category fluency - fruits	-0.02744	0.26223	0.9167	-0.35286	0.15738	0.02544*	0.88326	0.32909	0.00755**	1.16352	0.52488	0.02715*	1.40524	0.51609	0.00673**	0.59495	0.53521	0.2669	0.83629	0.2829	0.00328**
Boston naming	-0.08338	0.05706	0.14461	-0.11186	0.03413	0.00113**	0.10086	0.07227	0.16353	0.1106	0.11536	0.33819	0.12573	0.11374	0.26958	0.06385	0.11677	0.58483	0.1097	0.0624	0.07936
Perceptual orientation																					
Line orientation	-0.17266	0.13516	0.20212	-0.21223	0.08131	0.00935**	0.0418	0.1714	0.80742	-0.10239	0.27286	0.70766	0.01818	0.27051	0.94644	-0.10011	0.27721	0.71818	0.0743	0.1484	0.61683
Progressive matrices	-0.11	0.12238	0.35744	-0.19	0.07355	0.00939**	0.03892	0.15287	0.79918	-0.01	0.24339	0.95916	0.05949	0.24125	0.80535	-0.02	0.24727	0.92202	0.12824	0.1322	0.33271

	274			189						247						422					
Perceptual speed																					
Symbol digit modality	0.25958	0.50995	0.61098	-0.94998	0.30532	0.00198**	1.2742	0.64369	0.04838*	1.39514	1.02717	0.17509	0.95259	1.01931	0.35054	1.44021	1.04347	0.16822	1.00601	0.5578	0.07198
NART reading test	0.17898	0.33085	0.58879	-0.56467	0.1985	0.00465**	0.91008	0.41841	0.03016*	0.94772	0.66759	0.15643	1.03017	0.66113	0.11991	1.05224	0.67712	0.1209	0.76644	0.3622	0.03488*
Stroop color naming	0.20526	0.36329	0.57239	-0.63371	0.21633	0.00359**	0.83849	0.45481	0.06599	1.07865	0.73687	0.14404	1.33126	0.71791	0.06443	1.04714	0.73384	0.15439	0.85385	0.3959	0.03163*
Individual cognitive performance test scores																					
MMSE	-0.05735	0.09366	0.54066	-0.18819	0.05584	0.00082***	0.11418	0.11838	0.33529	0.01546	0.18852	0.93468	0.13055	0.18578	0.48261	0.12181	0.19136	0.52475	0.12223	0.1019	0.23089

Table S3. Logistic regression of metabolite marker panel-based RF score to discriminate NCI (converters) vs. NCI (non-converters) adjusting for gender, years of education, APOE ε4, BMI, fasting, and medications.

	Estimate	SE	*P*-value
**RF score**	7.828	1.036	**4.22E-14**
Gender (Male)	-0.303	0.439	0.49
Education	-0.009	0.059	0.874
APOE ε4-carrier	1.313	0.415	0.002
BMI (18.5-24.9)	1.001	1.11	0.367
BMI (25.0-29.9)	0.975	1.093	0.372
BMI (30.0 and Above)	0.31	1.122	0.783
Fasting (Unknown)	1.412	0.654	0.031
Fasting (Yes)	0.748	0.384	0.051
Supplement	0.277	0.594	0.641
Diabetes (insulin)	-13.983	753.838	0.985
diabetes (noninsulin)	0.039	0.585	0.947
Lipid lowering (non-statin)	0.995	0.579	0.086
Statin	0.312	0.335	0.353

Table S4. PDS of metabolic pathways differentially expressed in participants with cognitive decline.

**Brain samples**			
	**NCI**	**MCI**	**AD**
Primary bile acid biosynthesis (median [IQR])	0.67 [0.42, 0.78]	0.70 [0.66, 0.81]	0.82 [0.66, 0.92]*
Fatty acid biosynthesis (median [IQR])	0.05 [0.00, 0.16]	0.06 [0.02, 0.18]	0.12 [0.06, 0.42]**
Biosynthesis of unsaturated fatty acids (median [IQR])	0.05 [0.00, 0.17]	0.05 [0.01, 0.20]	0.15 [0.10, 0.36]***
**Serum samples**			
	**NCI**	**MCI/AD**	
Primary bile acid biosynthesis (median [IQR])	0.19 [0.03, 0.50]	0.33 [0.10, 0.57]##	
Fatty acid biosynthesis (median [IQR])	0.93 [0.73, 1.00]	0.97 [0.88, 1.00]##	
Biosynthesis of unsaturated fatty acids (median [IQR])	0.94 [0.83, 0.99]	0.97 [0.89, 1.00]###	

Abbreviations: NCI, cognitively normal; MCI, mild cognitive impairment; AD, Alzheimer's disease; IQR, interquartile range.

* P-value < 0.05, ** P-value < 0.01, *** P-value < 0.001, by Wilcoxon rank sum test comparing AD vs. NCI

# P-value < 0.05, ## P-value < 0.01, ### P-value < 0.001, by Wilcoxon rank sum test comparing MCI/AD vs. NCI

Table S5. Associations between identified metabolic pathways and cognitive performance tests adjusting for age, gender, years of education, APOE ε4, and BMI.

	Primary bile acid biosynthesis			Fatty acid biosynthesis			Biosynthesis of unsaturated fatty acids		
	Estimate	SE	P value	Estimate	SE	P value	Estimate	SE	P value
**Brain samples**									
Episodic memory									
cts_wli	-5.35169	2.28365	0.02141*	-8.57482	3.85677	0.02882*	-7.42135	3.9198	0.06168
cts_wlii	-2.80016	1.07415	0.01077*	-3.93915	1.83009	0.03416*	-3.56867	1.85565	0.05777
cts_ebmt	-1.42389	1.15437	0.22057	-4.38166	1.86604	0.02104*	-2.74907	1.917	0.15499
Working memory									
cts_doperf	-0.60812	0.63757	0.34274	-2.54812	1.04544	0.01676*	-1.63571	1.07116	0.13026
Semantic memory									
cts_animals	-2.39605	2.48878	0.33815	-8.25113	4.02025	0.04291*	-5.95725	4.14319	0.1538
Individual cognitive performance test scores									
cts_mmse30	-4.32828	2.43761	0.079	-8.96136	3.73328	0.01833*	-6.48538	3.85335	0.09565
**Serum samples**									
Episodic memory									
cts_wli	-1.11615	0.84391	0.18663	-4.02043	1.25558	0.00146**	-5.51627	1.70236	0.00128**
cts_wlii	-0.84784	0.42853	0.04846*	-1.5319	0.64237	0.01749*	-2.33573	0.86975	0.0075**
Semantic memory									
cts_catflu	-3.06599	1.62018	0.05906	-7.34202	2.43036	0.00266**	-8.92506	3.30223	0.00713**
cts_fruits	-1.37965	0.88192	0.11841	-3.73033	1.32296	0.00501**	-4.54141	1.79708	0.01183*
cts_animals	-1.68634	0.91413	0.06571	-3.6117	1.37421	0.00887**	-4.38365	1.86635	0.01925*

* *P*-value < 0.05, ** *P*-value < 0.01, *** *P*-value < 0.001

Table S6. Logistic regression of metabolic pathway panel-based RF score to discriminate NCI (converters) vs. NCI (non-converters) adjusting for gender, years of education, APOE ε4, BMI, fasting, and medications.

	Estimate	SE	*P*-value
**RF score**	5.629	0.847	**3.04E-11**
Gender (Male)	0.102	0.389	0.792
Education	-0.029	0.053	0.585
APOE ε4-carrier	0.942	0.372	0.011
BMI (18.5-24.9)	1.088	1.041	0.296
BMI (25.0-29.9)	1.38	1.036	0.183
BMI (30.0 and Above)	0.686	1.057	0.516
Fasting (Unknown)	1.231	0.591	0.037
Fasting (Yes)	0.285	0.341	0.403
Supplement	0.06	0.589	0.919
Diabetes (insulin)	-14.804	702.56	0.983
diabetes (noninsulin)	-0.271	0.544	0.619
Lipid lowering (non-statin)	1.109	0.574	0.053
Statin	0.338	0.313	0.28

Table S7. Levels of detected metabolites across clinical groups in brain samples.

Variable	NCI Mean (SD)	MCI/AD Mean (SD)
CA	0.23 (1.24)	0.30 (0.91)
CDCA	1.41 (1.03)	1.50 (1.08)
GCA	1.31 (1.03)	0.79 (0.97)
GCDCA	2.00 (1.13)	1.89 (0.90)
TaMCA	-0.57 (1.00)	-0.71 (1.07)
TbMCA	-0.37 (0.97)	-0.38 (0.95)
TCA	0.46 (1.55)	0.07 (1.11)
TCDCA	0.51 (1.54)	0.05 (1.37)
X12_ketoLCA	-0.05 (1.42)	0.06 (1.19)
X12_DHCA	-0.12 (1.21)	0.15 (1.09)
X3_DHCA	-0.79 (0.97)	-0.63 (0.87)
X6_ketoLCA	-0.77 (0.97)	-0.82 (1.00)
X7_12_diketoLCA	-0.53 (1.15)	-0.41 (1.31)
X7_ketoLCA	0.12 (1.14)	0.40 (0.83)
ACA	0.12 (1.31)	0.09 (1.11)
alloLCA	-0.33 (1.35)	0.17 (1.24)
apoCA	0.25 (1.85)	0.34 (1.13)
bMCA	-0.65 (0.90)	-0.60 (0.89)
bUCA	-1.17 (1.02)	-1.07 (0.88)
DCA	1.83 (1.41)	2.36 (1.20)
dehydroLCA	-0.21 (1.11)	0.20 (1.15)
DHCA	-0.80 (1.09)	-0.62 (0.99)
GDCA	0.85 (1.06)	1.04 (0.95)
GDHCA	-1.61 (1.14)	-1.45 (0.94)
GHCA	-0.79 (0.91)	-0.77 (0.81)
GHDCA	-1.32 (1.15)	-1.17 (0.99)
GLCA	-0.88 (1.01)	-0.49 (0.99)
GUDCA	-0.22 (0.92)	-0.09 (0.82)
HCA	-0.62 (0.93)	-0.53 (0.66)
HDCA	-0.66 (1.33)	-0.41 (0.91)
LCA	1.83 (0.81)	2.05 (0.78)
LCA_3S	-0.55 (1.03)	-0.15 (0.96)
muroCA	-0.77 (1.00)	-0.19 (0.98)
NorDCA	-0.55 (0.88)	-0.28 (0.68)
TDCA	0.99 (1.14)	1.11 (1.08)
TDHCA	-1.17 (1.11)	-0.91 (0.88)
THDCA	-1.21 (1.05)	-1.05 (0.87)
TUDCA	-0.77 (0.93)	-0.69 (0.77)
TwMCA	-0.50 (1.00)	-0.51 (0.94)
UCA	-1.42 (0.81)	-1.21 (0.72)
UDCA	0.66 (1.18)	0.68 (0.94)
CA_CDCA	-1.08 (0.99)	-1.20 (0.86)

DCA_CA	1.54 (1.27)	2.20 (1.25)
GDCA_CA	0.36 (1.70)	0.74 (1.54)
GDCA_CDCA	-0.56 (1.60)	-0.46 (1.55)
UDCA_CDCA	-0.74 (1.09)	-0.78 (1.16)
GDCA_DCA	-1.35 (1.23)	-1.54 (1.07)
TDCA_DCA	-1.18 (1.60)	-1.42 (1.53)
GCDCA_CDCA	0.57 (1.76)	0.39 (1.49)
TCDCA_CDCA	-0.90 (2.15)	-1.45 (1.91)
GLCA_CDCA	-2.29 (1.76)	-2.08 (1.99)
GUDCA_UDCA	-0.88 (1.22)	-0.87 (0.95)
TUDCA_UDCA	-1.43 (1.46)	-1.46 (1.20)
X1_5_Anhydrosorbitol	5.88 (0.72)	6.05 (0.74)
X1_Methylhistidine	3.54 (0.56)	3.67 (0.56)
X2_Hydroxy_3_methylbutyric_acid	4.28 (0.76)	4.33 (0.76)
X2_Hydroxybutyric_acid	6.05 (0.75)	6.17 (0.91)
X2_Hydroxypyridine	8.34 (0.41)	8.49 (0.38)
X3_Aminoisobutanoic_acid	3.43 (0.88)	3.61 (0.82)
X3_Nitrotyrosine	5.14 (0.72)	5.05 (0.97)
X3_Oxoalanine	3.79 (0.48)	3.95 (0.44)
X3_Sulfinato_L_alaninate	6.10 (0.53)	6.07 (0.42)
X4_Hydroxycinnamic_acid	6.81 (0.61)	6.93 (0.64)
X4_Hydroxyphenylpyruvic_acid	3.61 (0.65)	3.88 (0.74)
Acetylglycine	2.06 (0.32)	2.10 (0.47)
Adenine	4.16 (0.62)	4.23 (0.62)
Adenosine	4.83 (0.77)	5.11 (0.55)
Allantoin	4.92 (0.42)	5.02 (0.38)
Allocystathionine	7.74 (0.81)	7.86 (1.00)
Allose	6.14 (0.47)	6.19 (0.67)
Alpha_Tocopherol	4.21 (0.43)	4.29 (0.42)
Aminoadipic_acid	6.05 (0.38)	6.04 (0.55)
Arachidic_acid	4.61 (0.45)	4.72 (0.47)
Arachidonic_acid	6.53 (0.40)	6.68 (0.38)
Behenic_acid	2.83 (0.50)	2.93 (0.40)
Benzoic_acid	4.48 (0.71)	4.65 (0.64)
Beta_Alanine	4.30 (0.44)	4.46 (0.34)
Caffeine	3.13 (0.74)	3.17 (0.65)
Caproic_acid	3.01 (0.52)	2.99 (0.51)
Carnosine	3.20 (0.53)	3.45 (0.57)
Citrulline	4.66 (0.61)	4.87 (0.50)
Creatine	8.43 (0.61)	8.60 (0.44)
Creatinine	8.53 (0.37)	8.66 (0.29)
Cytidine	3.07 (0.48)	3.25 (0.41)
Cytosine	2.05 (0.47)	2.23 (0.50)
D_2_Hydroxyglutaric_acid	4.98 (0.71)	5.15 (0.58)
Dehydroascorbic_acid	6.94 (0.36)	7.11 (0.37)

D_Fructose	4.26 (0.76)	4.41 (1.14)
D_Glucose	6.08 (0.71)	6.19 (0.83)
D_Mannose	5.02 (1.41)	5.29 (1.56)
Docosahexaenoic_acid	5.89 (0.38)	6.01 (0.39)
D_Ribose	7.05 (0.50)	6.91 (0.51)
D_Tagatose	8.25 (0.60)	8.36 (0.94)
D_Threitol	6.70 (0.48)	6.83 (0.61)
D_Xylitol	7.14 (0.38)	7.35 (0.44)
D_Xylose	6.34 (0.36)	6.44 (0.44)
Elaidic_acid	6.12 (0.69)	6.25 (0.65)
Erythrose	5.71 (0.34)	5.82 (0.32)
Fructose_1_phosphate	4.67 (0.92)	4.42 (1.14)
Fructose_6_phosphate	4.88 (0.83)	4.93 (1.08)
Fumaric_acid	5.83 (0.49)	6.01 (0.39)
Galactonic_acid	4.39 (0.63)	4.46 (0.70)
Gamma_Aminobutyric_acid	9.30 (0.31)	9.35 (0.35)
Gluconolactone	4.63 (0.67)	4.84 (0.85)
Glucose_6_phosphate	5.01 (1.12)	5.18 (1.00)
Glutaric_acid	3.81 (0.43)	3.85 (0.50)
Glyceric_acid	4.45 (0.44)	4.64 (0.54)
Glycerol	9.28 (0.33)	9.32 (0.44)
Glycerol_3_phosphate	8.39 (0.57)	8.60 (0.72)
Glycine	8.91 (0.37)	9.04 (0.33)
Glycolic_acid	5.42 (0.57)	5.67 (0.55)
Guanosine	4.96 (0.48)	5.12 (0.41)
Heptadecanoic_acid	4.27 (0.62)	4.31 (0.60)
Hexanoylcarnitine	3.53 (0.43)	3.55 (0.69)
Homocysteine	4.39 (0.61)	4.52 (0.75)
Homogentisic_acid	5.07 (0.35)	5.27 (0.36)
Hydroxyphenyllactic_acid	3.33 (0.75)	3.59 (0.47)
Hypoxanthine	8.57 (0.43)	8.68 (0.34)
Inosine	8.05 (0.54)	8.16 (0.46)
L_Alloisoleucine	6.85 (0.60)	7.01 (0.54)
L_Allothreonine	4.57 (0.61)	4.57 (0.63)
L_Alpha_aminobutyric_acid	5.50 (0.50)	5.66 (0.42)
L_Arabinose	6.20 (0.50)	6.35 (0.32)
L_Arabitol	6.48 (0.45)	6.55 (0.34)
L_Arginine	5.05 (0.61)	5.22 (0.56)
L_Asparagine	5.44 (0.72)	5.44 (0.51)
L_Aspartic_acid	9.16 (0.38)	9.17 (0.43)
L_Cysteine	7.79 (0.44)	7.92 (0.34)
L_Cystine	4.91 (0.78)	5.05 (0.69)
L_Glutamic_acid	9.24 (0.53)	8.99 (1.15)
L_Glutamine	9.84 (0.43)	9.95 (0.43)
L_Histidine	7.11 (0.56)	7.32 (0.43)

Linoleic_acid	2.61 (0.51)	2.80 (0.60)
L_Lactic_acid	10.82 (0.49)	10.88 (0.52)
L_Leucine	7.84 (0.50)	7.98 (0.50)
L_Lysine	7.73 (0.56)	7.92 (0.48)
L_Methionine	6.50 (0.50)	6.62 (0.43)
L_Phenylalanine	7.53 (0.52)	7.70 (0.60)
L_Proline	7.24 (0.59)	7.41 (0.48)
L_Serine	7.75 (0.46)	7.89 (0.38)
L_Sorbose	7.86 (0.61)	7.97 (0.96)
L_Threonine	6.84 (0.60)	7.05 (0.52)
L_Tyrosine	8.12 (0.56)	8.35 (0.59)
L_Valine	7.14 (0.60)	7.34 (0.54)
L_Xylonate	5.22 (0.47)	5.58 (0.61)
Mannitol	8.72 (0.48)	8.78 (0.66)
Methionine_sulfoxide	2.48 (0.44)	2.64 (0.37)
Methylcysteine	3.93 (0.46)	4.07 (0.38)
Methylmalonic_acid	4.39 (0.64)	4.32 (0.60)
MG160	1.90 (0.41)	2.08 (0.46)
MG_18_1_9Z_0_0_0_0	2.87 (0.51)	3.08 (0.52)
MG_18_2_9Z_12Z_0_0_0_0	2.22 (0.48)	2.32 (0.56)
Myoinositol	10.27 (0.35)	10.25 (0.43)
Myristic_acid	5.09 (0.38)	5.22 (0.32)
N_Acetylglutamic_acid	5.16 (0.48)	5.30 (0.41)
N_Acetyl_L_aspartic_acid	9.50 (0.24)	9.58 (0.22)
Niacinamide	6.51 (0.33)	6.59 (0.32)
Oleic_acid	6.23 (0.49)	6.37 (0.42)
O_Phosphoethanolamine	8.92 (0.35)	8.99 (0.33)
Oxalic_acid	5.90 (0.72)	5.89 (0.83)
Palmitic_acid	9.03 (0.34)	9.20 (0.30)
Palmitoleic_acid	4.13 (0.43)	4.40 (0.47)
Pantothenic_acid	5.45 (0.61)	5.51 (0.61)
Pelargonic_acid	3.25 (0.59)	3.27 (0.48)
Pentadecanoic_acid	3.54 (0.60)	3.68 (0.41)
Petroselinic_acid	7.43 (0.42)	7.60 (0.37)
Phosphate	9.42 (0.73)	9.52 (0.56)
Picolinic_acid	4.95 (0.43)	5.03 (0.41)
Putrescine	3.64 (0.55)	3.69 (0.58)
Pyroglutamic_acid	10.65 (0.25)	10.75 (0.30)
Pyrophosphate	5.99 (0.45)	6.06 (0.52)
Pyruvic_acid	5.43 (0.60)	5.62 (0.59)
Rhamnose	2.61 (0.48)	2.68 (0.46)
Ribitol	2.09 (0.57)	2.34 (0.55)
Ribonolactone	5.76 (0.98)	5.65 (0.94)
Sarcosine	5.81 (0.71)	5.89 (0.88)
Sorbitol	6.04 (1.43)	6.27 (1.73)

Spermidine	5.31 (0.71)	5.49 (0.55)
Stearic_acid	9.20 (0.35)	9.36 (0.31)
Succinic_acid	6.51 (0.55)	6.54 (0.59)
Sucrose	2.57 (0.73)	2.86 (0.83)
Taurine	8.10 (0.59)	8.22 (0.47)
Threonic_acid	6.41 (0.38)	6.53 (0.39)
Uracil	4.95 (0.62)	5.02 (0.51)
Urea	6.62 (0.86)	6.76 (1.10)
Uric_acid	3.94 (0.78)	4.06 (0.83)
Uridine	3.80 (0.48)	3.99 (0.43)
Xanthine	6.56 (0.40)	6.57 (0.35)
Xanthosine	1.98 (0.46)	2.09 (0.45)

Abbreviations: NCI, cognitively normal; MCI, mild cognitive impairment; AD, Alzheimer's disease; SD, standard deviation; CA, Cholate; CDCA, Chenodeoxycholate; GCA, Glycocholate; GCDCA, Glycochenodeoxycholate; TαMCA, Tauro-α-muricholate; TβMCA, Tauro-β-muricholate; TCA, Taurocholate; TCDCA, Taurochenodeoxycholate; 12-ketoLCA, 12-Ketolithocholate; 12-DHCA, 12-dehydrocholate; 3-DHCA, 3-dehydrocholate; 6-ketoLCA, 6-Ketolithocholate; 7-12-diketoLCA, 7-12-diketolithocholate; 7-ketoLCA, 7-Ketolithocholate; ACA, Apocholate; alloLCA, Allolithocholate; apoCA, Apocholate; βMCA, β-muricholate; βUCA, β-ursocholate; DCA, Deoxycholate; dehydroLCA, Dehydrolithocholate; DHCA, Dehydrocholate; GDCA, Glycodeoxycholate; GDHCA, glycodehydrocholate; GHCA, Glycohyocholate; GHDCA, Glycohyodeoxycholate; GLCA, Glycolithocholate; GUDCA, Glycoursodeoxycholate; HCA, Hyocholate; HDCA, Hyodeoxycholate; LCA, Lithocholate; LCA-3S, Lithocholate-3S; muroCA, Murocholate; NorDCA, Nordeoxycholate; TDCA, Tautodeoxycholate; TDHCA, Taurodehydrocholate; THDCA, Taurohyodeoxycholi acid; TUDCA, Tauroursodeoxycholate; TωMCA, Tauro-ω-muricholate; UCA, Ursocholate; UDCA, Ursodeoxycholate.

Individual bile acid was adjusted by the total bile acids concentration (i.e., % of total bile acids).

All abundance values were log10 transformed.

Table S8. Levels of detected metabolites across clinical groups in serum samples.

Variable	NCI Mean (SD)	MCI/AD Mean (SD)
CA	0.17 (1.39)	-0.09 (1.49)
CDCA	1.07 (1.09)	1.02 (1.27)
GCA	1.80 (0.75)	1.71 (0.86)
GCDCA	3.18 (0.46)	3.10 (0.68)
TCA	-0.09 (1.25)	-0.19 (1.58)
TCDCA	0.99 (0.97)	0.95 (1.05)
TaMCA	-1.89 (1.59)	-1.87 (1.57)
TbMCA	-3.95 (1.36)	-3.93 (1.39)
TDCA	0.73 (1.23)	0.84 (1.08)
GDCA	2.51 (0.85)	2.58 (0.84)
GLCA	-0.19 (0.96)	0.05 (0.91)
DCA	2.40 (0.96)	2.36 (1.21)
12_ketoLCA	-0.54 (1.07)	-0.53 (0.99)
3_DHCA	-3.70 (1.43)	-3.72 (1.39)
7_ketoLCA	-1.93 (1.21)	-1.98 (1.35)
alloLCA	-1.85 (1.55)	-1.95 (1.75)
apoCA	-1.28 (1.32)	-1.39 (1.31)
dehydroLCA	-1.87 (1.06)	-2.08 (1.14)
GHCA	-1.66 (1.04)	-1.71 (1.21)
GUDCA	0.84 (0.94)	0.71 (0.98)
HCA	-1.94 (1.06)	-2.07 (1.25)
HDCA	-2.07 (1.05)	-2.16 (1.13)
isoLCA	-0.79 (1.17)	-0.95 (1.16)
LCA	-0.35 (0.94)	-0.42 (0.83)
LCA_3S	-1.14 (1.00)	-1.23 (1.07)
muroCA	-3.39 (0.91)	-3.34 (1.08)
NorDCA	-2.22 (1.12)	-2.27 (1.18)
TDHCA	-5.21 (1.29)	-5.38 (1.40)
THDCA	-5.05 (0.89)	-5.31 (1.10)
TUDCA	-2.64 (1.30)	-2.74 (1.46)
TwMCA	-3.76 (0.98)	-3.83 (0.97)
UCA	-3.69 (1.37)	-3.66 (1.29)
UDCA	-0.44 (1.22)	-0.58 (1.58)
bUCA	-4.32 (1.44)	-4.48 (1.52)
bUDCA	1.03 (1.30)	0.73 (1.26)
CA_CDCA	-0.90 (0.98)	-1.11 (1.06)
DCA_CA	2.23 (1.58)	2.59 (1.57)
GDCA_CA	2.34 (1.86)	2.66 (1.98)
GDCA_CDCA	1.43 (1.64)	1.56 (1.74)
UDCA_CDCA	-1.51 (1.20)	-1.60 (1.43)
GDCA_DCA	0.11 (0.87)	0.21 (0.96)
TDCA_DCA	-1.67 (1.39)	-1.52 (1.47)
GCDCA_CDCA	2.10 (1.25)	2.08 (1.50)

TCDCA_CDCA	-0.08 (1.69)	-0.07 (1.89)
GLCA_CDCA	-1.28 (1.71)	-1.19 (1.90)
GUDCA_UDCA	1.28 (1.07)	1.28 (1.43)
TUDCA_UDCA	-2.19 (1.57)	-2.17 (1.93)
Dimethylglycine	-0.11 (0.53)	-0.12 (0.46)
X2_Hydroxypyridine	-0.06 (0.40)	-0.00 (0.20)
Pyruvic_acid	-0.90 (1.13)	-0.84 (0.95)
L_Lactic_acid	-0.11 (0.53)	-0.04 (0.60)
Caproic_acid	-0.05 (0.43)	-0.06 (0.36)
Hexanoylcarnitine	-0.22 (0.47)	-0.17 (0.44)
Glycolic_acid	-0.01 (0.25)	-0.00 (0.33)
L_Alanine	-0.22 (0.46)	-0.13 (0.35)
Hydroxylamine	0.01 (0.46)	0.03 (0.50)
X2_Hydroxybutyric_acid	-0.15 (0.64)	-0.14 (0.63)
Oxalic_acid	-0.10 (0.68)	-0.07 (0.68)
Sarcosine	-0.34 (0.98)	-0.31 (0.88)
m_Cresol	-0.34 (0.71)	-0.10 (0.71)
X2_Hydroxy_3_methylbutyric_acid	-0.14 (0.57)	-0.21 (0.50)
L_Alpha_aminobutyric_acid	-0.12 (0.55)	-0.21 (0.51)
Methylmalonic_acid	-0.09 (0.41)	-0.08 (0.38)
L_Valine	-0.29 (0.54)	-0.23 (0.32)
Glyceraldehyde	-0.16 (0.43)	-0.17 (0.40)
Urea	-0.14 (0.61)	-0.17 (0.51)
Benzoic_acid	-0.04 (0.50)	-0.14 (0.42)
Phosphoric_acid	-0.21 (0.69)	-0.26 (0.60)
L_Leucine	-0.23 (0.44)	-0.22 (0.38)
Glycerol	-0.16 (0.53)	-0.21 (0.45)
Phosphate	-0.01 (0.61)	0.07 (0.55)
L_Isoleucine	-0.23 (0.62)	-0.29 (0.51)
L_Alloisoleucine	-0.23 (0.43)	-0.25 (0.39)
L_Proline	-0.23 (0.66)	-0.14 (0.55)
Glycine	-0.23 (0.42)	-0.19 (0.30)
Picolinic_acid	-0.09 (0.53)	-0.16 (0.49)
Nicotinic_acid	-0.08 (0.30)	-0.09 (0.30)
Glyceric_acid	-0.03 (0.42)	-0.05 (0.42)
Fumaric_acid	-0.12 (0.35)	-0.10 (0.40)
Pelargonic_acid	-0.14 (0.84)	-0.25 (0.77)
L_Serine	-0.19 (0.29)	-0.24 (0.33)
Indoxyl_sulfate	-0.14 (0.68)	0.12 (0.84)
L_Pipecolic_acid	-0.15 (0.65)	-0.20 (0.65)
L_Threonine	-0.20 (0.34)	-0.24 (0.33)
Glutaric_acid	-0.07 (0.57)	-0.12 (0.52)
Methylcysteine	-0.35 (0.57)	-0.40 (0.50)
Beta_Alanine	-0.19 (0.45)	-0.17 (0.52)
Decanoylcarnitine	-0.09 (0.47)	-0.12 (0.47)

Erythrose	-0.14 (0.55)	-0.10 (0.42)
Aminomalonic_acid	-0.38 (0.58)	-0.35 (0.49)
D_Threitol	-0.90 (1.04)	-0.70 (0.78)
L_Methionine	-0.49 (0.59)	-0.52 (0.57)
L_Aspartic_acid	-0.22 (0.35)	-0.21 (0.35)
Pyroglutamic_acid	-0.20 (0.36)	-0.17 (0.21)
X2_Phenylglycine	-0.25 (0.61)	-0.24 (0.57)
Gamma_Aminobutyric_acid	-0.15 (0.43)	-0.12 (0.35)
Threonic_acid	0.12 (0.65)	0.16 (0.56)
L_Cysteine	-0.14 (0.57)	-0.08 (0.66)
Creatinine	-0.36 (0.54)	-0.33 (0.55)
Threonic_acid	-0.23 (0.50)	-0.24 (0.52)
D_2_Hydroxyglutaric_acid	-0.18 (0.45)	-0.23 (0.45)
Phenylpyruvic_acid	-0.13 (0.41)	-0.14 (0.34)
X3_Hydroxybutyric_acid	-0.21 (0.54)	-0.16 (0.57)
L_Arginine	-0.25 (0.52)	-0.24 (0.56)
L_Glutamic_acid	-0.26 (0.46)	-0.27 (0.43)
L_Phenylalanine	-0.20 (0.31)	-0.21 (0.26)
N_Acetyl_L_aspartic_acid	0.15 (0.71)	0.17 (0.60)
Homocysteine	-0.31 (0.51)	-0.20 (0.53)
L_Asparagine	-0.15 (0.35)	-0.10 (0.37)
D_Xylose	-0.12 (0.36)	-0.07 (0.33)
L_Arabinose	-0.16 (0.52)	-0.07 (0.41)
Taurine	-0.27 (0.46)	-0.16 (0.51)
Suberic_acid	-0.17 (0.42)	-0.27 (0.55)
Ribonolactone	-0.37 (0.60)	-0.37 (0.59)
Aminoadipic_acid	-0.07 (0.52)	-0.23 (0.56)
L_Arabitol	-0.24 (0.39)	-0.16 (0.36)
Rhamnose	-0.21 (0.47)	-0.09 (0.46)
Ribitol	-0.30 (0.57)	-0.29 (0.74)
Putrescine	-0.31 (0.69)	-0.18 (0.68)
Glycerol_3_phosphate	-0.05 (0.53)	-0.06 (0.53)
L_Glutamine	-0.22 (0.41)	-0.16 (0.28)
Azelaic_acid	-0.18 (0.43)	-0.27 (0.49)
O_Phosphoethanolamine	-0.40 (0.84)	-0.24 (0.80)
Hypoxanthine	-0.11 (0.41)	-0.03 (0.53)
X1_Methylhistidine	-0.30 (0.89)	-0.23 (0.83)
Citrulline	-0.28 (0.35)	-0.23 (0.29)
Myristic_acid	-0.17 (0.49)	-0.37 (0.47)
X1_5_Anhydrosorbitol	-0.26 (0.60)	-0.28 (0.57)
X3_Hydroxyanthranilic_acid	-0.16 (0.76)	-0.09 (0.80)
Fructose_6_phosphate	-0.07 (0.61)	-0.05 (0.56)
L_Sorbose	-0.05 (0.62)	-0.04 (0.57)
D_Fructose	-0.20 (0.37)	-0.14 (0.32)
Gluconolactone	-0.18 (0.44)	-0.27 (0.42)

D_Mannose	0.07 (0.64)	0.08 (0.52)
Allose	-0.14 (0.99)	-0.19 (0.83)
L_Lysine	-0.42 (0.49)	-0.38 (0.39)
L_Histidine	-0.33 (0.37)	-0.35 (0.39)
D_Glucose	-0.13 (1.02)	-0.09 (1.09)
D_Galactose	-0.22 (0.39)	-0.19 (0.24)
L_Tyrosine	-0.24 (0.40)	-0.23 (0.31)
Mannitol	-0.49 (0.88)	-0.28 (0.84)
D_Glucuronic_acid	-0.36 (0.66)	-0.15 (0.63)
Sorbitol	-0.10 (0.51)	-0.03 (0.53)
Pantothenic_acid	-0.33 (0.57)	-0.34 (0.61)
Palmitoleic_acid	-0.37 (0.90)	-0.76 (0.92)
Galactonic_acid	-0.19 (0.50)	-0.10 (0.44)
Gluconic_acid	-0.18 (0.49)	-0.16 (0.47)
Palmitic_acid	-0.13 (0.48)	-0.24 (0.35)
X3_Indolepropionic_acid	0.06 (0.76)	-0.02 (0.64)
X5_Hydroxylysine	-0.17 (0.53)	-0.18 (0.56)
Uric_acid	-0.23 (0.62)	-0.18 (0.59)
Myoinositol	-0.25 (0.51)	-0.06 (0.43)
Heptadecanoic_acid	-0.09 (0.32)	-0.16 (0.31)
Linoleic_acid	-0.12 (0.47)	-0.25 (0.47)
Petroselinic_acid	-0.26 (0.76)	-0.56 (0.74)
Oleic_acid	-0.12 (0.55)	-0.31 (0.59)
Elaidic_acid	-0.10 (0.58)	-0.29 (0.59)
Stearic_acid	-0.10 (0.39)	-0.14 (0.30)
Spermidine	-0.02 (0.50)	-0.04 (0.42)
L_Cystine	-0.15 (0.48)	-0.12 (0.49)
Arachidonic_acid	-0.00 (0.42)	-0.05 (0.46)
Arachidic_acid	-0.12 (0.39)	-0.12 (0.29)
Uridine	-0.12 (0.47)	-0.19 (0.48)
Carnosine	-0.20 (0.56)	-0.18 (0.41)
Docosahexaenoic_acid	0.01 (0.57)	-0.08 (0.56)
MG160	-0.15 (0.26)	-0.13 (0.21)
Inosine	0.07 (0.65)	0.11 (0.65)
Behenic_acid	-0.12 (0.61)	-0.14 (0.49)
Sucrose	-0.11 (0.66)	-0.05 (0.69)
Xanthosine	0.16 (0.67)	0.22 (0.59)
MG182	-0.10 (0.48)	-0.22 (0.51)
Glycerol_1_octadecanoate	-0.14 (0.30)	-0.13 (0.17)
D_Maltose	0.03 (0.51)	0.07 (0.51)
Tetracosanoic_acid	-0.13 (0.70)	-0.14 (0.61)
Hexacosanoic_acid	-0.04 (0.20)	-0.02 (0.15)
Alpha_Tocopherol	-0.19 (0.39)	-0.18 (0.43)

Abbreviations: NCI, cognitively normal; MCI, mild cognitive impairment; AD, Alzheimer's disease; SD, standard deviation; CA, Cholate; CDCA, Chenodeoxycholate; GCA, Glycocholate; GCDCA, Glycochenodeoxycholate; TCDCA, Taurochenodeoxycholate; TαMCA, Tauro-α-muricholate; TβMCA, Tauro-β-muricholate; TDCA, Taurodeoxycholate; GDCA, Glycodeoxycholate; GLCA, Glycolithocholate; DCA, Deoxycholate; 12-ketoLCA, 12-Ketolithocholate; 3-DHCA, 3-dehydrocholate; 7-ketoLCA, 7-Ketolithocholate; alloLCA, Allolithocholate; apoCA, Apocholate; dehydroLCA, Dehydrolithocholate; GHCA, Glycohyocholate; GUDCA, Glycoursodeoxycholate; HCA, Hyocholate; HDCA, Hyodeoxycholate; LCA, Lithocholate; isoLCA, Isolithocholate; LCA-3S, Lithocholate-3S; muroCA, Murocholate; NorDCA, Nordeoxycholate; TDHCA, Taurodehydrocholate; THDCA, Taurohyodeoxycholi acid; TUDCA, Tauroursodeoxycholate; TωMCA, Tauro-ω-muricholate; UCA, Ursocholate; UDCA, Ursodeoxycholate; βUCA, β-ursocholate; βUDCA, β-ursodeoxycholate.

Individual bile acid was adjusted by the total bile acids concentration (i.e., % of total bile acids).

All abundance values were log10 transformed.

Table S9. PDS of mapped metabolic pathways across clinical groups in brain samples.

Metabolic Pathways	NCI Mean (SD)	MCI/AD Mean (SD)
Carbon fixation in photosynthetic organisms	0.26 (0.29)	0.29 (0.29)
Purine metabolism	0.27 (0.27)	0.41 (0.32)
Nicotinate and nicotinamide metabolism	0.17 (0.17)	0.24 (0.23)
Thiamine metabolism	0.21 (0.24)	0.33 (0.30)
Glutathione metabolism	0.25 (0.25)	0.37 (0.30)
Glyoxylate and dicarboxylate metabolism	0.17 (0.16)	0.26 (0.22)
Pantothenate and CoA biosynthesis	0.25 (0.24)	0.30 (0.25)
Glycine serine and threonine metabolism	0.14 (0.17)	0.20 (0.16)
Carbon fixation pathways in prokaryotes	0.19 (0.22)	0.27 (0.25)
Methane metabolism	0.15 (0.17)	0.26 (0.24)
Arginine biosynthesis	0.28 (0.31)	0.44 (0.35)
Alanine aspartate and glutamate metabolism	0.19 (0.18)	0.24 (0.20)
Cyanoamino acid metabolism	0.13 (0.18)	0.20 (0.19)
Pyrimidine metabolism	0.17 (0.19)	0.28 (0.22)
Cysteine and methionine metabolism	0.18 (0.21)	0.20 (0.18)
Arginine and proline metabolism	0.21 (0.23)	0.28 (0.24)
Ascorbate and aldarate metabolism	0.26 (0.29)	0.34 (0.30)
Glycolysis Gluconeogenesis	0.19 (0.18)	0.19 (0.18)
Taurine and hypotaurine metabolism	0.32 (0.19)	0.39 (0.19)
Citrate cycle	0.19 (0.22)	0.27 (0.25)
Valine leucine and isoleucine biosynthesis	0.14 (0.21)	0.21 (0.23)
Butanoate metabolism	0.18 (0.18)	0.24 (0.21)
Pyruvate metabolism	0.22 (0.22)	0.28 (0.25)
Pentose phosphate pathway	0.28 (0.14)	0.39 (0.21)
Phenylalanine metabolism	0.13 (0.16)	0.22 (0.22)
Monobactam biosynthesis	0.14 (0.18)	0.23 (0.22)
Tyrosine metabolism	0.16 (0.18)	0.29 (0.24)
beta Alanine metabolism	0.25 (0.24)	0.39 (0.27)
Propanoate metabolism	0.24 (0.25)	0.29 (0.25)
Lysine degradation	0.20 (0.21)	0.22 (0.24)
Valine leucine and isoleucine degradation	0.20 (0.17)	0.24 (0.20)
Lysine biosynthesis	0.22 (0.24)	0.29 (0.30)
Fatty acid biosynthesis	0.09 (0.13)	0.18 (0.21)
Porphyrin and chlorophyll metabolism	0.20 (0.21)	0.26 (0.22)
Histidine metabolism	0.22 (0.22)	0.30 (0.23)
Butirosin and neomycin biosynthesis	0.18 (0.17)	0.17 (0.18)
Pentose and glucuronate interconversions	0.27 (0.15)	0.39 (0.23)
Starch and sucrose metabolism	0.12 (0.15)	0.15 (0.19)
Galactose metabolism	0.21 (0.15)	0.25 (0.18)
Amino sugar and nucleotide sugar metabolism	0.12 (0.15)	0.16 (0.20)
Glycerolipid metabolism	0.17 (0.26)	0.32 (0.33)
Streptomycin biosynthesis	0.18 (0.20)	0.18 (0.19)

Sulfur metabolism	0.21 (0.23)	0.28 (0.25)
Primary bile acid biosynthesis	0.59 (0.30)	0.72 (0.22)
Tropane piperidine and pyridine alkaloid biosynthesis	0.10 (0.20)	0.16 (0.19)
Phenylalanine tyrosine and tryptophan biosynthesis	0.16 (0.21)	0.28 (0.27)
Glucosinolate biosynthesis	0.10 (0.18)	0.17 (0.20)
Novobiocin biosynthesis	0.10 (0.19)	0.19 (0.21)
Ubiquinone and other terpenoid quinone biosynthesis	0.13 (0.17)	0.26 (0.24)
Fructose and mannose metabolism	0.19 (0.18)	0.23 (0.21)
Styrene degradation	0.15 (0.17)	0.31 (0.25)
Biosynthesis of unsaturated fatty acids	0.11 (0.14)	0.19 (0.20)

Abbreviations: NCI, cognitively normal; MCI, mild cognitive impairment; AD, Alzheimer's disease; SD, standard deviation;

Table S10. PDS of mapped metabolic pathways across clinical groups in serum samples.

	NCI Mean (SD)	MCI/AD Mean (SD)
Carbon fixation in photosynthetic organisms	0.30 (0.28)	0.32 (0.31)
Purine metabolism	0.28 (0.14)	0.29 (0.18)
Nicotinate and nicotinamide metabolism	0.79 (0.22)	0.78 (0.24)
Thiamine metabolism	0.23 (0.25)	0.22 (0.24)
Glutathione metabolism	0.49 (0.13)	0.51 (0.13)
Glyoxylate and dicarboxylate metabolism	0.39 (0.17)	0.38 (0.16)
Pantothenate and CoA biosynthesis	0.86 (0.14)	0.85 (0.16)
Glycine serine and threonine metabolism	0.65 (0.14)	0.65 (0.13)
Nitrogen metabolism	0.45 (0.11)	0.46 (0.11)
Methane metabolism	0.54 (0.13)	0.53 (0.13)
Arginine biosynthesis	0.53 (0.11)	0.53 (0.11)
Alanine aspartate and glutamate metabolism	0.48 (0.12)	0.48 (0.13)
Cyanoamino acid metabolism	0.57 (0.11)	0.58 (0.09)
Pyrimidine metabolism	0.41 (0.15)	0.42 (0.13)
Cysteine and methionine metabolism	0.30 (0.17)	0.28 (0.16)
Arginine and proline metabolism	0.73 (0.28)	0.72 (0.28)
Ascorbate and aldarate metabolism	0.12 (0.15)	0.17 (0.19)
Glycolysis Gluconeogenesis	0.09 (0.12)	0.12 (0.16)
Taurine and hypotaurine metabolism	0.23 (0.24)	0.26 (0.24)
Valine leucine and isoleucine biosynthesis	0.87 (0.18)	0.88 (0.12)
Butanoate metabolism	0.53 (0.24)	0.51 (0.23)
Pyruvate metabolism	0.10 (0.12)	0.12 (0.15)
Pentose phosphate pathway	0.53 (0.22)	0.54 (0.20)
Phenylalanine metabolism	0.50 (0.14)	0.49 (0.13)
Monobactam biosynthesis	0.63 (0.19)	0.63 (0.18)
Tyrosine metabolism	0.14 (0.17)	0.16 (0.18)
beta Alanine metabolism	0.59 (0.09)	0.59 (0.09)
Propanoate metabolism	0.41 (0.06)	0.42 (0.09)
Lysine degradation	0.71 (0.27)	0.76 (0.25)
Valine leucine and isoleucine degradation	0.49 (0.12)	0.51 (0.09)
Lysine biosynthesis	0.60 (0.11)	0.60 (0.11)
Fatty acid biosynthesis	0.85 (0.18)	0.90 (0.14)
Porphyrin and chlorophyll metabolism	0.86 (0.18)	0.87 (0.15)
Histidine metabolism	0.63 (0.13)	0.63 (0.11)
Pentose and glucuronate interconversions	0.26 (0.14)	0.28 (0.15)
Starch and sucrose metabolism	0.15 (0.18)	0.20 (0.20)
Galactose metabolism	0.36 (0.12)	0.39 (0.13)
Amino sugar and nucleotide sugar metabolism	0.35 (0.20)	0.36 (0.20)
Glycerolipid metabolism	0.84 (0.21)	0.86 (0.20)
Primary bile acid biosynthesis	0.28 (0.27)	0.36 (0.28)

Tropane piperidine and pyridine alkaloid biosynthesis	0.53 (0.15)	0.54 (0.13)
Phenylalanine tyrosine and tryptophan biosynthesis	0.89 (0.17)	0.90 (0.13)
Glucosinolate biosynthesis	0.88 (0.18)	0.90 (0.11)
Fructose and mannose metabolism	0.41 (0.07)	0.41 (0.10)
Biosynthesis of unsaturated fatty acids	0.89 (0.14)	0.93 (0.11)

Abbreviations: NCI, cognitively normal; MCI, mild cognitive impairment; AD, Alzheimer's disease; SD, standard deviation;

Table S11. Levels of identified metabolites in samples with both brain and blood metabolomics data

**Brain samples**			
	**NCI**	**MCI**	**AD**
GLCA % (median [IQR])	0.44 [0.24, 0.85]	0.50 [0.23, 0.95]	0.88 [0.54, 1.26]*
DCA/CA (median [IQR])	4.98 [1.90, 9.87]	9.14 [5.46, 16.29]	8.08 [3.49, 29.53]*
Petroselinic acid (median [IQR])	1773.24 [1336.51, 2180.72]	1805.46 [1495.15, 2366.08]	2183.78 [1768.03, 2804.06]**
Linoleic acid (median [IQR])	14.93 [11.39, 18.22]	13.60 [9.84, 20.35]	20.36 [16.88, 32.13]***
Myristic acid (median [IQR])	168.36 [133.77, 210.46]	173.05 [136.37, 201.52]	209.63 [178.55, 250.68]**
Palmitic acid (median [IQR])	9447.81 [7175.45, 10634.46]	8762.88 [7642.75, 10873.54]	10651.09 [9135.51, 12702.42]**
Palmitoleic acid (median [IQR])	65.58 [49.05, 78.58]	75.00 [59.59, 97.51]	89.73 [65.83, 132.13]**
**Serum samples**			
	**NCI**	**MCI/AD**	
GLCA % (median [IQR])	0.95 [0.57, 1.41]	1.14 [0.61, 1.61]	
DCA/CA (median [IQR])	12.97 [2.88, 27.57]	19.24 [5.64, 51.86]	
Petroselinic acid (median [IQR])	0.52 [0.32, 1.53]	0.37 [0.25, 0.85]	
Linoleic acid (median [IQR])	0.72 [0.52, 1.37]	0.65 [0.48, 1.00]	
Myristic acid (median [IQR])	0.72 [0.54, 1.36]	0.59 [0.40, 1.00]#	
Palmitic acid (median [IQR])	0.81 [0.59, 1.26]	0.74 [0.56, 0.96]	
Palmitoleic acid (median [IQR])	0.50 [0.22, 1.58]	0.38 [0.19, 0.73]	

* P-value < 0.05, ** P-value < 0.01, *** P-value < 0.001, by Wilcoxon rank sum test comparing AD vs. NCI

\# P-value < 0.05, by Wilcoxon rank sum test comparing MCI/AD vs. NCI

Abbreviations: NCI, cognitively normal; MCI, mild cognitive impairment; AD, Alzheimer's disease; IQR, interquartile range.

Table S12. Mixed effects model of metabolite marker panel-based RF score adjusting for age, gender, years of education, APOE ε4, and BMI.

	Estimate	Standard Errors	*P*-value
cogn_global	-0.520	0.139	<0.001
cogn_ep	-0.475	0.185	0.011
cogn_po	-0.340	0.218	0.120
cogn_ps	-0.685	0.255	0.008
cogn_se	-0.598	0.177	<0.001
cogn_wo	-0.532	0.241	0.028

Table S13. Associations between identified metabolites/ratio and cognitive performance domains adjusting for age, gender, years of education, APOE ε4, and BMI.

	GLCA			DCA/CA			Petroselinic acid			Linoleic acid			Myristic acid			Palmitic acid			Palmitoleic acid		
	Estimate	SE	P value	Estimate	SE	P value	Estimate	SE	P value	Estimate	SE	P value	Estimate	SE	P value	Estimate	SE	P value	Estimate	SE	P value
**Brain samples**																					
cogn_ep	-0.04749	0.11898	0.69077	-0.22	0.09187	0.01883*	-0.74783	0.33437	0.02794*	-0.34401	0.2376	0.15133	-0.48176	0.35655	0.18022	-0.91776	0.3742	0.01623*	-0.75642	0.27912	0.00814**
cogn_wo	-0.02064	0.08101	0.79948	-0.17973	0.06257	0.00502**	-0.01955	0.21977	0.92932	-0.23846	0.15176	0.11944	-0.15344	0.2364	0.51784	-0.11998	0.25711	0.64184	-0.19918	0.18465	0.28346
cogn_se	-0.09291	0.09875	0.34948	-0.17015	0.07714	0.03016*	-0.18629	0.26713	0.48751	-0.20567	0.18988	0.28188	-0.16524	0.28918	0.56926	-0.33509	0.3133	0.28792	-0.22656	0.23327	0.33426
cogn_po	0.00856	0.07754	0.91242	-0.12367	0.06206	0.04988*	0.00967	0.20836	0.96309	0.04966	0.14778	0.7378	0.05389	0.22746	0.81335	0.0307	0.24532	0.90075	0.11942	0.18291	0.5158
cogn_ps	-0.04254	0.11059	0.70148	-0.24389	0.08603	0.00576**	-0.24476	0.29682	0.41196	-0.28695	0.20719	0.16977	-0.52276	0.3172	0.10313	-0.37186	0.34812	0.28853	-0.44623	0.24534	0.07255
**Serum samples**																					
cogn_ep	-0.05384	0.03661	0.14215	-0.05471	0.022	0.01324*	0.05969	0.04642	0.19918	0.0386	0.07396	0.60206	0.06288	0.07289	0.38874	0.03102	0.07512	0.67983	0.06743	0.03992	0.09189
cogn_wo	0.03624	0.0339	0.28562	-0.03371	0.02043	0.09973	0.07146	0.04276	0.09541	0.1174	0.06801	0.08501	0.12518	0.06702	0.06246	0.02978	0.06928	0.66751	0.09468	0.03666	0.01013*
cogn_se	-0.00499	0.02969	0.86664	-0.05414	0.01778	0.00246**	0.07119	0.03748	0.05819	0.0656	0.05986	0.27369	0.11441	0.05894	0.0529	0.02496	0.06072	0.68123	0.07387	0.03234	0.02285*
cogn_po	-0.04448	0.03315	0.18034	-0.0631	0.01987	0.0016**	0.01261	0.04167	0.76237	-0.01778	0.06633	0.78882	0.01221	0.06576	0.85275	-0.01928	0.06739	0.7749	0.03174	0.03605	0.37904
cogn_ps	0.02255	0.03949	0.56824	-0.07691	0.02362	0.00122**	0.11212	0.04973	0.02464*	0.11957	0.07942	0.13291	0.10639	0.07876	0.17746	0.12852	0.08066	0.11181	0.09169	0.04309	0.03389*

* *P*-value < 0.05, ** *P*-value < 0.01, *** *P*-value < 0.001

Table S14. P values and Q values of identified metabolites across clinical groups in brain samples using ordinal regression.

Variable	P value	Q value
GLCA	0.030	0.048
DCA_CA	0.005	0.023
Linoleic_acid	0.017	0.037
Myristic_acid	0.012	0.031
Palmitic_acid	0.002	0.018
Palmitoleic_acid	0.001	0.015
Petroselinic_acid	0.012	0.031

Table S15. P values and Q values of identified metabolites across clinical groups in serum samples using logistic regression.

Variable	P value	Q value
GLCA	0.015	0.127
DCA_CA	0.029	0.159
Myristic_acid	<0.001	0.006
Palmitoleic_acid	<0.001	0.006
Palmitic_acid	0.034	0.161
Linoleic_acid	0.009	0.090
Petroselinic_acid	<0.001	0.006

Table S16. P values and Q values of identified pathway across clinical groups in brain samples using ordinal regression.

Variable	P value	Q value
Fatty acid biosynthesis	0.003	0.003
Primary bile acid biosynthesis	0.009	0.007
Biosynthesis of unsaturated fatty acids	0.003	0.003

Table S17. P values and Q values of identified pathway across clinical groups in serum samples using logistic regression.

Variable	P value	Q value
Fatty acid biosynthesis	0.002	0.002
Primary bile acid biosynthesis	0.009	0.007
Biosynthesis of unsaturated fatty acids	0.005	0.004

Table S18. P values identified pathway across clinical groups in serum samples adjusting for other potential confounders using logistic regression.

Variable	adjusted_Variable	adjusted_P_value_Serum
GLCA	fasting	0.059
GLCA	supplement_rx	0.033
GLCA	diabetes_insulin_rx	0.047
GLCA	diabetes_noninsulin_rx	0.035
GLCA	lipid_lowering_nonstatin_rx	0.035
GLCA	statin_rx	0.030
DCA_CA	fasting	0.037
DCA_CA	supplement_rx	0.016
DCA_CA	diabetes_insulin_rx	0.020
DCA_CA	diabetes_noninsulin_rx	0.018
DCA_CA	lipid_lowering_nonstatin_rx	0.014
DCA_CA	statin_rx	0.012
Linoleic_acid	fasting	0.007
Linoleic_acid	supplement_rx	<0.001
Linoleic_acid	diabetes_insulin_rx	<0.001
Linoleic_acid	diabetes_noninsulin_rx	<0.001
Linoleic_acid	lipid_lowering_nonstatin_rx	<0.001
Linoleic_acid	statin_rx	<0.001
Myristic_acid	fasting	0.038
Myristic_acid	supplement_rx	0.003
Myristic_acid	diabetes_insulin_rx	0.003
Myristic_acid	diabetes_noninsulin_rx	0.003
Myristic_acid	lipid_lowering_nonstatin_rx	0.004
Myristic_acid	statin_rx	0.003
Palmitic_acid	fasting	0.087
Palmitic_acid	supplement_rx	0.015
Palmitic_acid	diabetes_insulin_rx	0.014
Palmitic_acid	diabetes_noninsulin_rx	0.015
Palmitic_acid	lipid_lowering_nonstatin_rx	0.016
Palmitic_acid	statin_rx	0.015
Palmitoleic_acid	fasting	0.062
Palmitoleic_acid	supplement_rx	0.003
Palmitoleic_acid	diabetes_insulin_rx	0.002
Palmitoleic_acid	diabetes_noninsulin_rx	0.003
Palmitoleic_acid	lipid_lowering_nonstatin_rx	0.004
Palmitoleic_acid	statin_rx	0.004

Petroselinic_acid	fasting	0.004
Petroselinic_acid	supplement_rx	<0.001
Petroselinic_acid	diabetes_insulin_rx	<0.001
Petroselinic_acid	diabetes_noninsulin_rx	<0.001
Petroselinic_acid	lipid_lowering_nonstatin_rx	<0.001
Petroselinic_acid	statin_rx	<0.001

fasting: fasting status at time of blood draw
supplement_rx: supplement usage in last 2 weeks
diabetes_insulin_rx: insulin usage in last 2 weeks
diabetes_noninsulin_rx: non-insulin diabetes medications usage in last 2 weeks
lipid_lowering_nonstatin_rx: non-statin lipid lowering medications usage in last 2 weeks
statin_rx: statin usage in last 2 weeks
